# Integrated transcriptomic and metabolomic analysis to elucidate key genes and signaling pathways involved in the promotion of periodontitis by hypertension

**DOI:** 10.1038/s41598-026-48279-8

**Published:** 2026-04-13

**Authors:** Haiqing Liao, Cuiping Li, Zhiqing Feng, Chenrui Fan, Jialei Xu, Ruiyi Guo, Yulian Pang, Lufei Wang, Xiaoping Su

**Affiliations:** 1 Department of Periodontics and Oral Medicine, College and Hospital of Stomatology, Guangxi Medical University, No.10, Shuangyong Road, Nanning, 530021 Guangxi China; 2 Guangxi Key Laboratory of Oral and Maxillofacial Rehabilitation and Reconstruction, College and Hospital of Stomatology, Guangxi Medical University, No.10, Shuangyong Road, Nanning, 530021 Guangxi China; 3 Guangxi Health Commission Key Laboratory of Prevention and Treatment for Oral Infectious Diseases, College and Hospital of Stomatology, Guangxi Medical University, No.10, Shuangyong Road, Nanning, 530021 Guangxi China

**Keywords:** Periodontitis, Hypertension, Transcriptome, Metabolome, Arginine, Macrophage, Dental diseases, Oral diseases, Periodontitis

## Abstract

To identify key genes and metabolites that promote the progression of periodontitis in the context of hypertension through combined transcriptomic and metabolomic analyses. Rat models of periodontitis and periodontitis combined with hypertension were established, with healthy rats serving as the control group. Periodontal tissues from each group were collected for transcriptomic and metabolomic sequencing. Subsequently, differential genes and metabolites were analyzed in the periodontitis with hypertension group compared to the control group, as well as in the periodontitis with hypertension group compared to the periodontitis group. Gene Ontology and KEGG enrichment analyses were performed, and a joint analysis of differential genes and metabolites was conducted. A total of 631 differential mRNAs and 67 differential metabolites were identified in the periodontitis with hypertension group compared to the control group. The differential genes and metabolites were mainly enriched in the pathways of phenylalanine, tyrosine, and tryptophan biosynthesis, phenylalanine metabolism, arginine biosynthesis, and arginine and proline metabolism. In the periodontitis with hypertension group compared to the periodontitis group, 135 differential mRNAs and 24 differential metabolites were identified. The differential genes and metabolites were primarily enriched in the pathways of arginine biosynthesis, cysteine and methionine metabolism, and biosynthesis of amino acids. Citrulline significantly suppresses the production of reactive oxygen species and the expression of the inflammatory cytokine interleukin-8 (IL-8) in gingival fibroblasts. Hypertension may promote the development of periodontitis by altering interferon-inducible genes and the arginine metabolism-related pathway. Citrulline represents a potential therapeutic option for periodontitis.

**Supplementary Information:**

The online version contains supplementary material available at 10.1038/s41598-026-48279-8.

## Introduction

Periodontitis is a multifactorial inflammatory disease characterized primarily by the destruction of the supportive structures of the teeth^1,2^. The etiology of periodontitis is complex, involving a combination of microbial, host, and environmental factors. The imbalance between the plaque biofilms and the host is regarded as a key factor in the progression of periodontitis, leading to chronic inflammation and tissue destruction^3,4^. Additionally, periodontitis is influenced by overall health status, lifestyle factors such as smoking, and dietary patterns, which may exacerbate the inflammatory response associated with the disease^3,5^.The implications of periodontitis extend beyond oral health, significantly affecting systemic health. Current research has established associations between periodontitis and various systemic diseases, including diabetes, respiratory diseases, and cardiovascular conditions, notably hypertension^6^. Inflammatory mediators released during periodontal inflammation can enter the bloodstream, resulting in systemic inflammation and potentially triggering the onset of these diseases^6^. Elevated levels of inflammatory markers associated with periodontitis (such as CRP, IL-6, TNF-α)^7,8^, alterations in endothelial function^9,10^, and changes in the coagulation system^11^ may contribute to the development of hypertension.

Simultaneously, the relationship between periodontitis and hypertension is bidirectional, with hypertension also influencing the progression of periodontitis. A large body of epidemiologic studies has indicated that patients with hypertension exhibit a higher prevalence and more severe cases of periodontitis. Research by Aguilera et al. suggests that hypertension may induce alterations in the microcirculation of gingival tissues, leading to increased ischemia and inflammation^12^. Findings by Del Pinto et al. corroborate this, highlighting a significant association between active gingival inflammation and hypertension, potentially mediated by inflammatory markers such as reactive oxygen species (ROS) and tumor necrosis factor (TNF)^13^. One of the primary mechanisms through which hypertension affects periodontitis is the enhancement of inflammatory responses. Studies have demonstrated that hypertensive patients exhibit elevated levels of inflammatory markers, such as C-reactive protein (CRP) and interleukin-6 (IL-6), which are known factors contributing to the destruction of periodontal tissues^7,14^. Bonato et al. have provided evidence that hypertension promotes the inflammatory process associated with periodontitis, resulting in increased bone loss^15^. Similarly, Rosa et al. found that hypertension exacerbates the activation of immune cells, leading to increased periodontal inflammation and subsequent tissue damage^14^. Moreover, the relationship between hypertension and periodontitis may also involve vascular changes. Hypertension can lead to endothelial dysfunction, which in turn affects the blood flow to periodontal tissues, exacerbating periodontal disease^15^.

Hypertension significantly influences the occurrence and progression of periodontitis, potentially due to the interaction of common inflammatory responses, risk factors, and inflammatory mediators; however, the specific underlying mechanisms remain unclear. This study aims to construct models of periodontitis and hypertension in rats and, through transcriptomic and metabolomic analyses, to identify key genes and metabolites involved in the impact of hypertension on the progression of periodontitis. The findings are intended to provide more effective references for the prevention and treatment of periodontitis in the context of hypertension.

## Results

### Differential gene analysis between periodontitis with hypertension group and control group

**Fig. 1:**
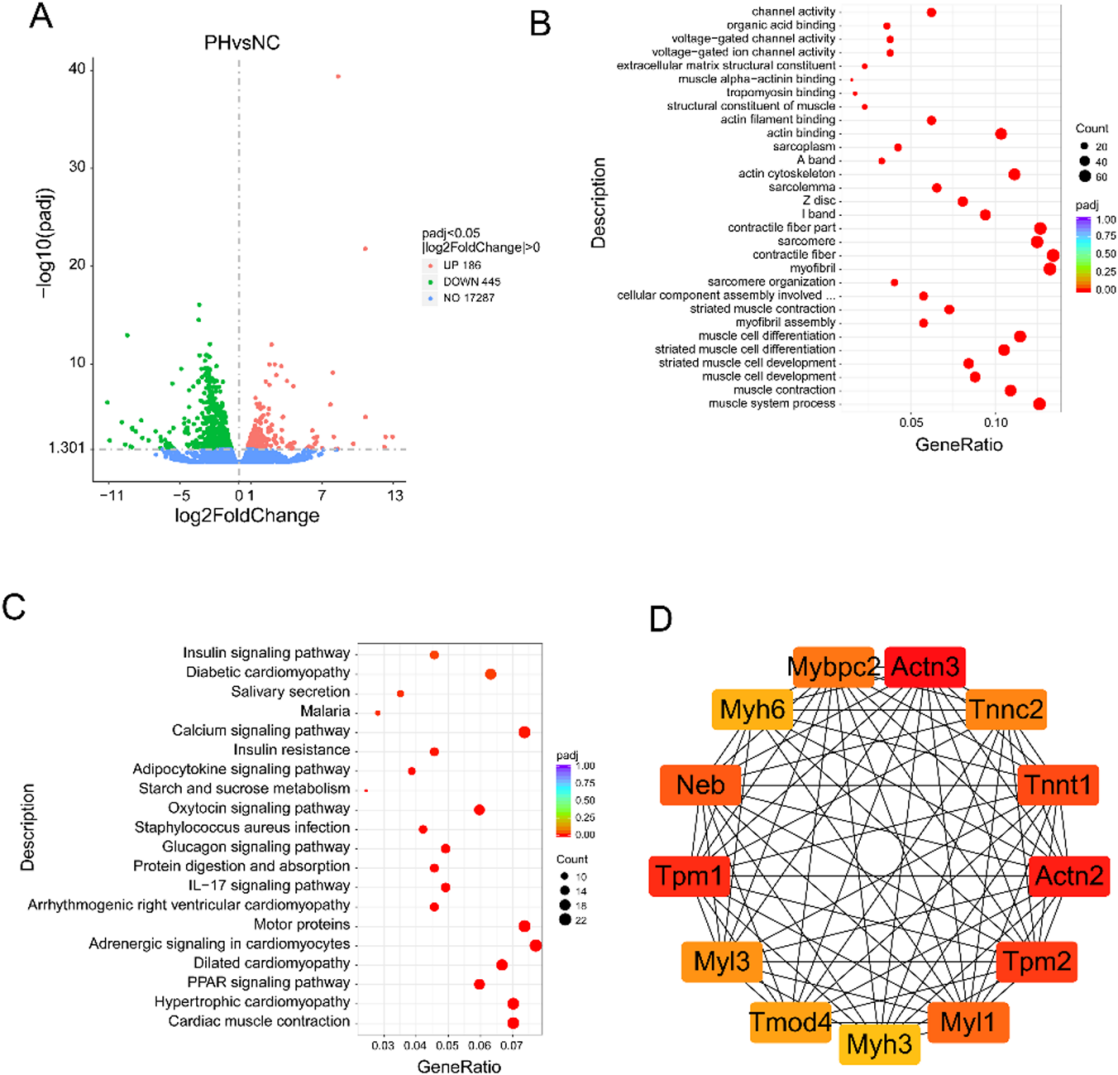
Differential mRNA analysis of hypertension combined with periodontitis group compared to control group. (**A**) Volcano plot of differential mRNA. Green dots indicate downregulated genes, red dots indicate upregulated genes, blue dots indicate no differential gene. (**B**) GO enrichment analysis of differential mRNA; (**C**) KEGG enrichment analysis of differential mRNA; (**D**) protein-protein interaction analysis of differential mRNA. In the volcano plot, green dots represent downregulated genes, red dots indicate upregulated genes, and blue dots denote non-differentially expressed genes. The protein-protein interaction (PPI) network was constructed using the STRING database, and hub genes were identified using the cytoHubba plugin in Cytoscape. PH, periodontitis with hypertension; NC, normal control (healthy group).

To investigate the key genes and signaling pathways associated with the occurrence of periodontitis combined with hypertension, we constructed a rat model of periodontitis with hypertension and performed whole transcriptome sequencing. The sequencing results showed that, compared to the control group, 186 mRNAs were upregulated and 445 were downregulated in the periodontal tissues of rats with periodontitis and hypertension (Fig. 1A). GO enrichment analysis revealed that the differentially expressed genes were primarily enriched in biological processes such as muscle system process and muscle contraction, cellular components such as myofibril and contractile fiber, and molecular functions such as actin binding and actin filament binding (Fig. 1B). KEGG enrichment analysis indicated that the differentially expressed genes were mainly enriched in signaling pathways including Cardiac muscle contraction, Hypertrophic cardiomyopathy, PPAR signaling pathway, and Dilated cardiomyopathy (Fig. 1C, Table S1). To screen for key genes related to the occurrence of periodontitis combined with hypertension, an interaction network among the differentially expressed genes was established using the online tool STRING, and key genes were identified using the Cytoscape software plugin cytoHubba. The results suggested that genes such as Actn3, Actn2, Tpm1, Tpm2, and Tnnt1 may play significant regulatory roles in the occurrence of hypertension combined with periodontitis (Fig. 1D).

### Differential lncRNA analysis in periodontitis with hypertension compared to healthy controls

**Fig. 2:**
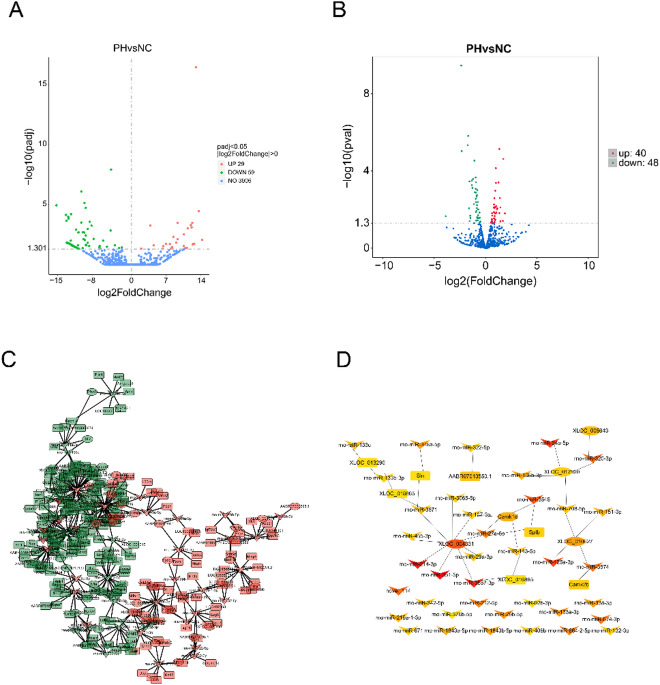
WTS Analysis of differential lncRNA, miRNA, and RNA in hypertension combined with periodontitis group compared to control group. (**A**) Volcano plot of differential lncRNA; (**B**) volcano plot of differential miRNA; (**C**) interaction network diagram of mRNA, lncRNA, and miRNA; (**D**) core gene selection using cytoHubba. In the volcano plot, green dots represent downregulated genes, red dots indicate upregulated genes, and blue dots correspond to non-differentially expressed genes. In the network diagram, ellipses represent mRNAs, rounded rectangular nodes denote lncRNAs, and V-shaped nodes correspond to miRNAs.

Compared to the control group, a total of 29 lncRNAs were significantly upregulated and 59 lncRNAs were significantly downregulated in the periodontal tissues of rats with periodontitis combined with hypertension (Fig. 2A). We performed GO and KEGG enrichment analyses on the co-localized genes and co-expressed genes associated with the differentially expressed lncRNAs. The analysis results indicated that the co-expressed genes were primarily enriched in biological processes such as muscle cell development, striated muscle cell development, and muscle cell differentiation. In terms of cellular components, they were notably enriched in myofibrils, contractile fibers, and sarcomeres. Additionally, the molecular functions included cytoskeletal protein binding, actin binding, and substrate-specific channel activity, as well as involvement in signal pathways such as cardiac muscle contraction, hypertrophic cardiomyopathy, dilated cardiomyopathy, and adrenergic signaling in cardiomyocytes (Fig.S1A, B). The co-localized genes associated with the differentially expressed lncRNAs were mainly enriched in biological processes such as positive regulation of lymphocyte-mediated immunity, positive regulation of adaptive immune response based on somatic processes, and positive regulation of T cell-mediated immunity. For cellular components, they were connected to keratin filaments, MHC protein complexes, and myosin complexes. Their molecular functions included structural molecule activity, and the enriched signaling pathways included type I diabetes mellitus, antigen processing and presentation, allograft rejection, and graft-versus-host disease (Fig. S1C, D).

### Differential miRNA analysis in periodontitis with hypertension compared to healthy controls

Compared to the control group, 40 miRNAs were upregulated and 48 miRNAs were downregulated in the periodontal tissues of rats with periodontitis combined with hypertension(Fig. 2B). GO and KEGG enrichment analysis were performed on the target genes of differentially expressed miRNAs. The analysis results showed that the target genes were mainly enriched in biological processes such as B cell activation and humoral immune response mediated by circulating immunoglobulin, cellular components such as immunoglobulin complex, circulating and immunoglobulin complex, and molecular functions such as immunoglobulin receptor binding and antigen binding (Fig.S1E). They were also enriched in signaling pathways such as Pathways in cancer, beta-Alanine metabolism, Glutathione metabolism, and Calcium signaling pathway (Fig.S1F).

### Analysis of the interaction network among differentially expressed mRNA, lncRNA, and miRNA in periodontitis with hypertension compared to healthy controls

To identify the interactive relationships between differentially expressed transcripts in the periodontitis combined with hypertension group compared to the control group, a whole-genome association analysis was conducted using the WTS method. A regulatory network of lncRNA-miRNA-mRNA was constructed, and key genes and interaction relationships were screened using the MCC method in cytoHubba. The analysis results revealed that XLOC_004331, miR-3557-3p, and XLOC_016885, as well as XLOC_010627, miR-3574, and Camk2b exhibited closely interconnected interaction relationships(Fig. 2C, D).

### Differential metabolite analysis between periodontitis with hypertension group and control group

**Fig. 3:**
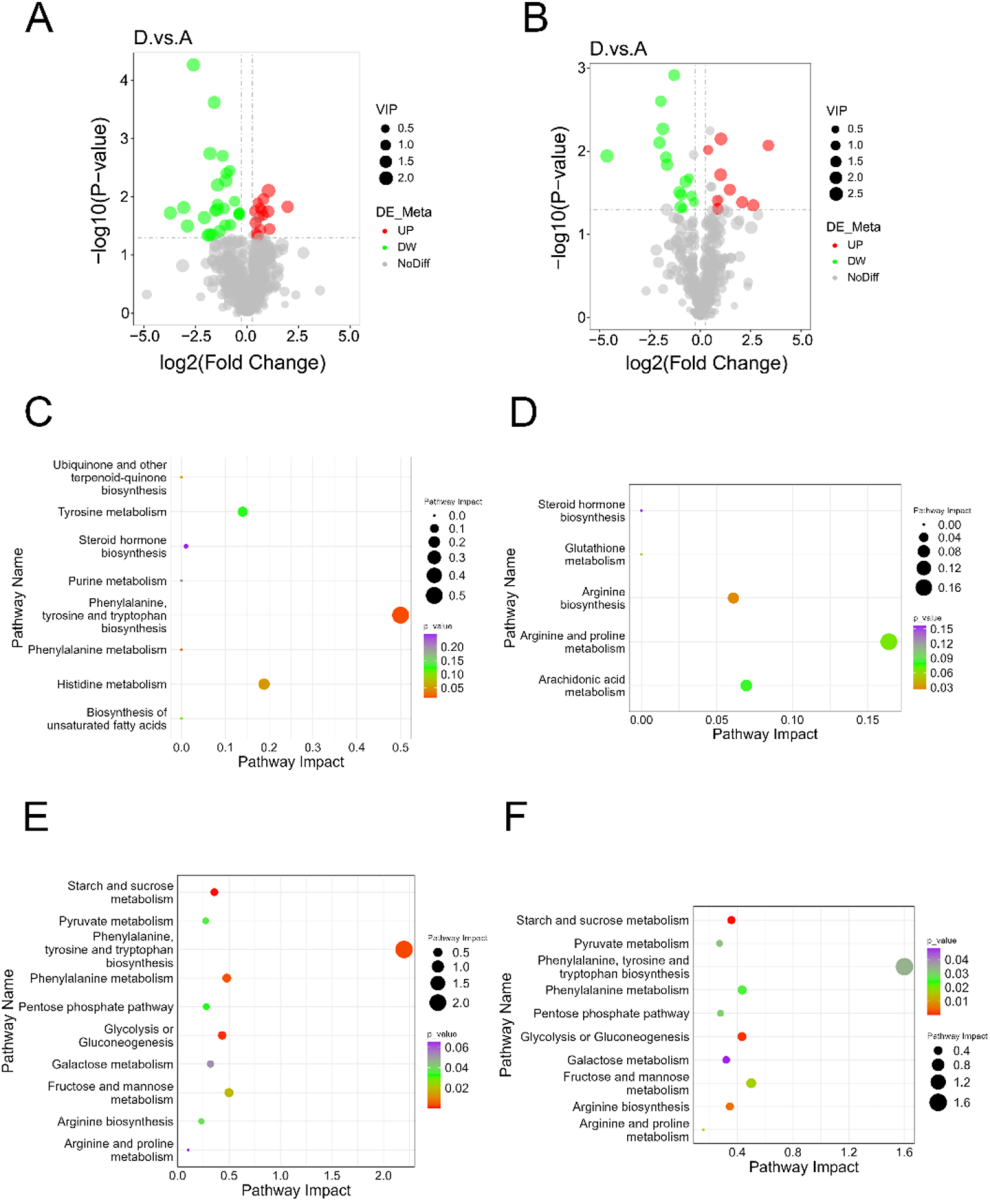
Analysis of differential metabolites and their correlation with differential mRNA in the hypertension and periodontitis group compared to the control group. (**A**) Volcano plot of differential metabolites in positive ion mode; (**B**) Volcano plot of differential metabolites in negative ion mode; (**C**) KEGG analysis of differential metabolites in positive ion mode; (**D**) KEGG analysis of differential metabolites in negative ion mode; (**E**) Co-enrichment analysis of KEGG pathways for differential metabolites and differential mRNA under cation mode; (**F**) Co-enrichment analysis of KEGG pathways for differential metabolites and differential mRNA under anion mode. In the volcano plot, green dots represent downregulated metabolites, red dots indicate upregulated metabolites, and gray dots denote non-differentially expressed metabolites, with dot size corresponding to the VIP value. D, periodontitis with hypertension group; A, control group. In the pathway enrichment plot, the dot size represents the pathway impact value, and the color gradient indicates the level of significance.

To investigate the changes in metabolites during the progression of periodontitis in conjunction with hypertension, we compared the metabolites in the periodontal tissues of rats with periodontitis and hypertension to those of healthy rats. Metabolomic data assessment involved the use of principal component analysis (PCA) and partial least square discriminant analysis (PLS-DA) (Fig.S2). The analysis revealed a total of 42 differential metabolites in the positive polarity mode, of which 15 were upregulated and 27 were downregulated (Fig. 3A). In the negative polarity mode, 25 differential metabolites were identified, with 10 upregulated and 15 downregulated (Fig. 3B). KEGG enrichment analysis indicated that the differential metabolites in the positive polarity mode were predominantly enriched in metabolic pathways such as phenylalanine, tyrosine, and tryptophan biosynthesis, phenylalanine metabolism, and histidine metabolism. In contrast, the metabolites in the negative polarity mode were mainly enriched in the Purine metabolism pathway (Fig. 3C, D, Table S2).

### Analysis of associations between differential genes and differential metabolites in periodontitis with hypertension compared to healthy controls

By constructing a correlation network diagram, we performed a joint analysis of differentially expressed transcripts and differential metabolites. The results indicated that, in the positive polarity mode, differentially expressed mRNAs such as AABR07050399.2, Lhcgr, Foxl2, and Mybpc2 were centrally located in the correlation network alongside differential metabolites including D-Homocysteine, HKK, Dextromethorphan hydrobromide, 6,7-dihydro-5 H-dibenzo[d, f][1,3]diazepin-6-one, Oleic acid, and 1,3-Dimethyluracil (Fig.S3A). Differential lncRNAs such as Cyp7b1, XLOC_034351, XLOC_011115, LOC102550455, and AABR07026377.1 also occupied central positions in the network with differential metabolites including HKK, 1-adamantyl(piperidino)methanone, Oleic acid, 6,7-dihydro-5 H-dibenzo[d, f][1,3]diazepin-6-one, and 6-(3-hydroxybutan-2-yl)-5-(hydroxymethyl)-4-methoxy-2 H-pyran-2-one (Fig.S3C). Additionally, differentially expressed miRNAs such as rno-miR-19b-3p, rno-miR-133a-5p, rno-miR-133a-3p, and rno-miR-133c were found to be centrally located in the network in relation to differential metabolites including L-Tyrosine, (3-Methoxy-4-hydroxyphenyl)ethylene glycol sulfate, Coumarin, 2-Hydroxycinnamic acid, N-Benzylformamide, D-Homocysteine, and 6-(3-hydroxybutan-2-yl)-5-(hydroxymethyl)-4-methoxy-2 H-pyran-2-one (Fig.S3E).

In the negative polarity mode, differentially expressed mRNAs such as Agbl1, Sdhd, Smtnl2, and Fbp2 were centrally located in the correlation network alongside differential metabolites including 15(S)-HpETE, N-Acetyl-L-phenylalanine, LysoPE 18:2, Ornithine, S-(Methyl)Glutathione, GW501516, and LPS 22:6 (Fig.S3 B). Additionally, differential lncRNAs such as AABR07026377.1, Cyp7b1, XLOC_031686, LOC102550455, and XLOC_004760 were also found to occupy central positions in the network, along with metabolites including GW501516, LPS 22:6, 15(S)-HpETE, S-(Methyl)Glutathione, Roquefortine C, and Ornithine (Fig.S3D). Furthermore, differentially expressed miRNAs such as rno-miR-542-3p, rno-miR-19b-3p, and rno-miR-133a-5p were identified as being centrally located in relation to differential metabolites, which included LPC 22:5, Roquefortine C, LPE 22:6, LPE 22:5, LPC 22:6, Ornithine, and LysoPE 18:2 (Fig.S3F).

KEGG enrichment analysis of differentially expressed mRNAs and metabolites revealed that in the positive polarity mode, L-Tyrosine co-enriched with tyrosine aminotransferase (Tat) and Got1 in the Phenylalanine, tyrosine and tryptophan biosynthesis pathway, as well as with Got1, Tat, and Ddc in the Phenylalanine metabolism pathway (Fig. 3E, Table S3). In the negative polarity mode, Ornithine was found to co-enrich with Nos1, LOC497963 (Nos2), and Got1 in the Arginine biosynthesis pathway, and with Nos1, LOC497963 (Nos2), Ckm, Ckmt2, and Got1 in the Arginine and proline metabolism pathway (Fig. 3F, Table S3).

### Differential gene analysis between periodontitis with hypertension group and periodontitis group

**Fig. 4:**
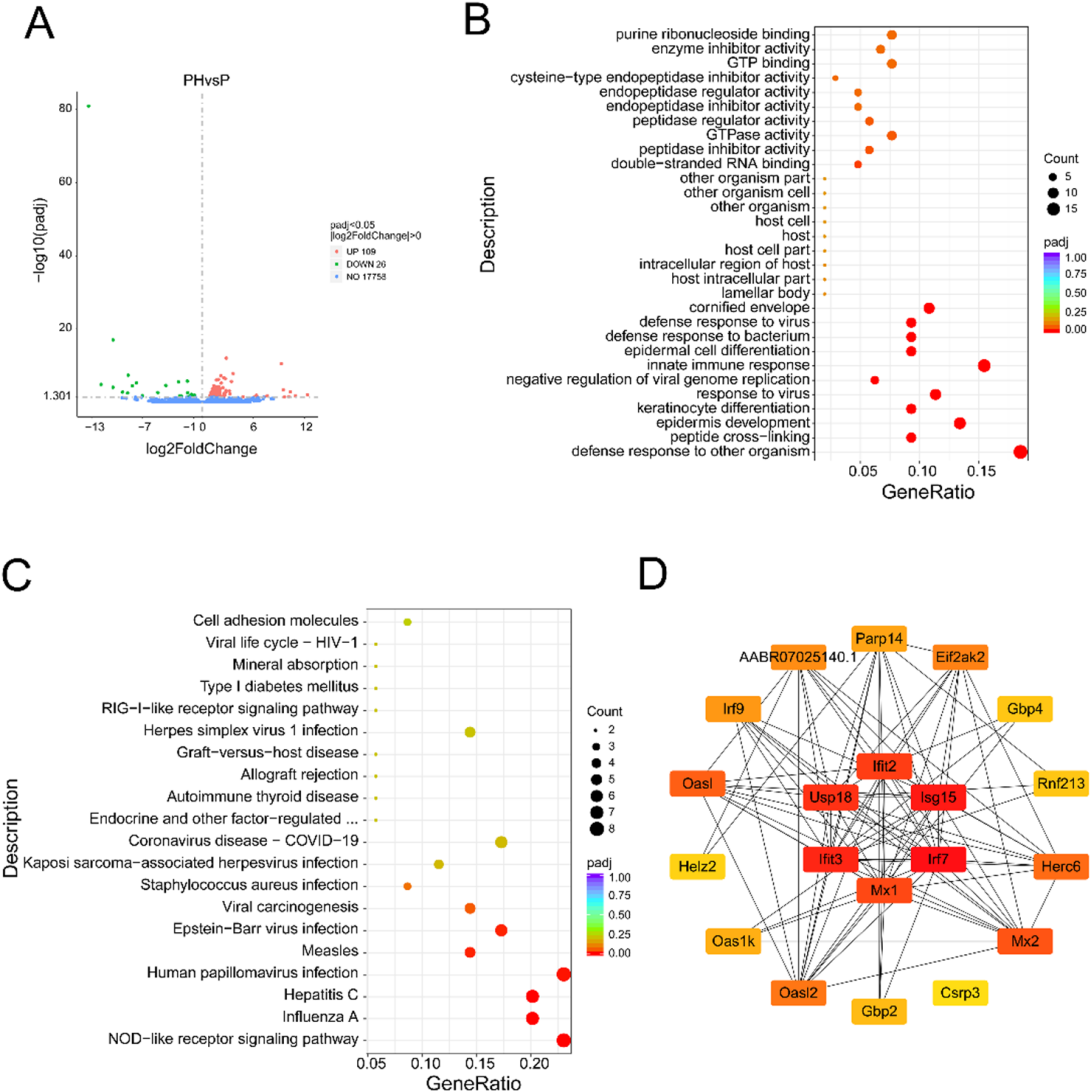
Differential mRNA analysis of hypertension combined with periodontitis group compared to periodontitis group. (**A**) Volcano plot of differential mRNA; (**B**) GO enrichment analysis of differential mRNA; (**C**) KEGG enrichment analysis of differential mRNA; (**D**) Protein-protein interaction analysis of differential mRNA. In the volcano plot, green dots represent downregulated genes, red dots indicate upregulated genes, and blue dots correspond to non-differentially expressed genes. The protein-protein interaction (PPI) network was constructed using the STRING database, and hub genes were identified using the cytoHubba plugin in CytoScape. PH, periodontitis with hypertension; P, periodontitis alone.

To explore the differences in mRNA expression between periodontitis complicated with hypertension and periodontitis alone, high-throughput sequencing was performed on tissue samples from both groups. The results revealed that 109 mRNA expressions were up-regulated and 26 mRNA expressions were down-regulated in the periodontitis complicated with hypertension group compared to the periodontitis group(Fig. 4A, Table S4). GO enrichment analysis showed that the differentially expressed genes were mainly involved in biological processes such as defense response to other organisms, peptide cross-linking, and epidermis development. In terms of cellular components, the differentially expressed genes were enriched in the cornified envelope. Molecular function analysis indicated functions such as double-stranded RNA binding and peptidase inhibitor activity (Fig. 4B). KEGG enrichment analysis revealed that the differentially expressed genes were mainly enriched in signaling pathways such as the NOD-like receptor signaling pathway, Influenza A, Hepatitis C, and Human papillomavirus infection (Fig. 4C). To identify key genes that contribute to the complexity of periodontitis in the context of hypertension, an interaction network among the differentially expressed genes was constructed using the online tool STRING, and key genes were identified using the CytoScape software plug-in cytoHubba. The results indicated that genes such as Usp18, lfit3, Isg15, Irf7, Ifit2, and Mx1 may play important regulatory roles in the development of periodontitis combined with hypertension (Fig. 4D).

### Differential lncRNA analysis in periodontitis with hypertension compared to periodontitis-only

**Fig. 5:**
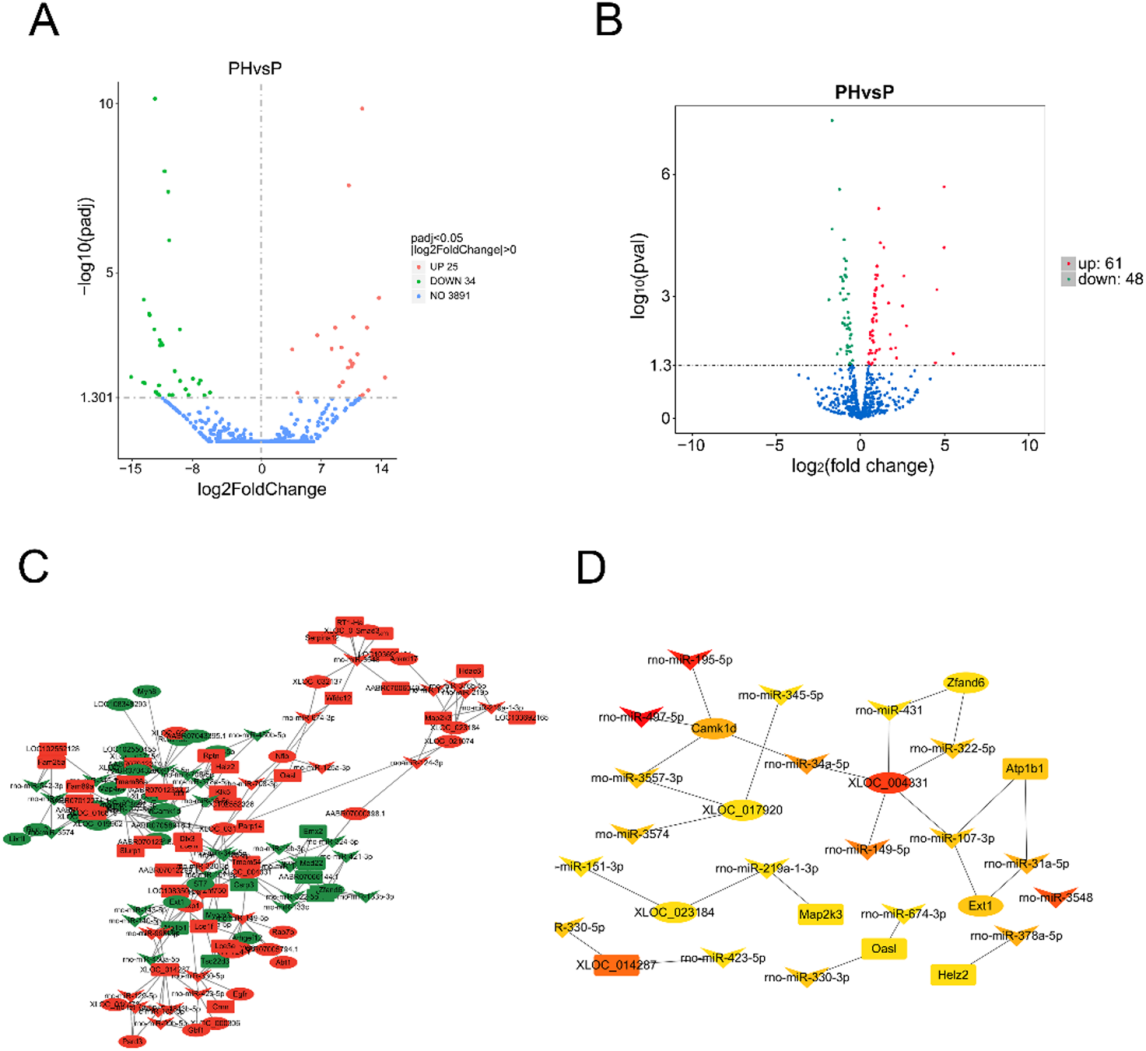
WTS Analysis of differential lncRNA, miRNA, and RNA in hypertension combined with periodontitis group compared to periodontitis group. (**A**) Volcano plot of differential lncRNA; (**B**) Volcano plot of differential miRNA; (**C**) Interaction network diagram of mRNA, lncRNA, and miRNA; (**D**) Core gene selection using cytoHubba. In the volcano plot, green dots represent downregulated genes, red dots indicate upregulated genes, and blue dots denote non-differentially expressed genes. In the network diagram, rounded rectangular nodes denote mRNAs, ellipses represent lncRNAs, and V-shaped nodes correspond to miRNAs.

Compared to the periodontitis group, the periodontitis combined with hypertension group exhibited upregulation of 25 lncRNAs and downregulation of 34 lncRNAs in the periodontal tissues of rats(Fig. 5A). GO and KEGG enrichment analyses were performed on the co-localized genes and co-expressed genes of the differentially expressed lncRNAs. The analysis results indicated that no significant enrichment was observed in either GO or KEGG pathways. (Fig.S4A, B).

The co-localized genes of the differentially expressed lncRNAs were mainly enriched in biological processes such as antigen processing and presentation of peptide antigen via MHC class Ib, antigen processing and presentation via MHC class Ib, and antigen processing and presentation of endogenous peptide antigen via MHC class Ib. In terms of cellular components, the enrichment was observed in keratin filament and external side of plasma membrane. In terms of molecular functions, the enrichment was observed in peptide antigen binding and antigen binding(Fig.S4C). The KEGG enrichment analysis indicated enrichment in signaling pathways such as Human papillomavirus infection, Endocytosis, Autoimmune thyroid disease, Allograft rejection, and Graft-versus-host disease (Fig.S4D).

### Differential miRNA analysis in periodontitis with hypertension compared to periodontitis-only

Compared to the periodontitis group, in the periodontitis combined with hypertension group of rats, 61 miRNAs were upregulated and 48 miRNAs were downregulated in periodontal tissues(Fig. 5B). The target genes of differentially expressed miRNAs were subjected to GO and KEGG enrichment analysis. The analysis results showed that the target genes were mainly enriched in biological processes such as B cell activation, leukocyte mediated immunity, phagocytosis, and recognition, cellular components including immunoglobulin complex and immunoglobulin complex, circulating, molecular functions like immunoglobulin receptor binding and antigen binding(Fig.S4E). However, no significantly enriched KEGG signaling pathways were identified (Fig.S4F).

### Analysis of the interaction network among differentially expressed mRNA, lncRNA, and miRNA in periodontitis with hypertension compared to periodontitis-only

In order to identify the differential RNA interactions between the group with periodontitis combined with hypertension and the group with periodontitis alone, a whole-genome association analysis was conducted using the WTS method. A lncRNA-miRNA-mRNA regulatory network was constructed, and key genes and interactions were screened using the MCC method of cytoHubba. The analysis revealed interactions between Ext1, miR-107-3p, and Atp1b1, as well as between XLOC_023184, miR-219a-1-3p, and Map2k3(Fig. 5C, D).

### Differential metabolite analysis between periodontitis with hypertension group and periodontitis group

**Fig. 6:**
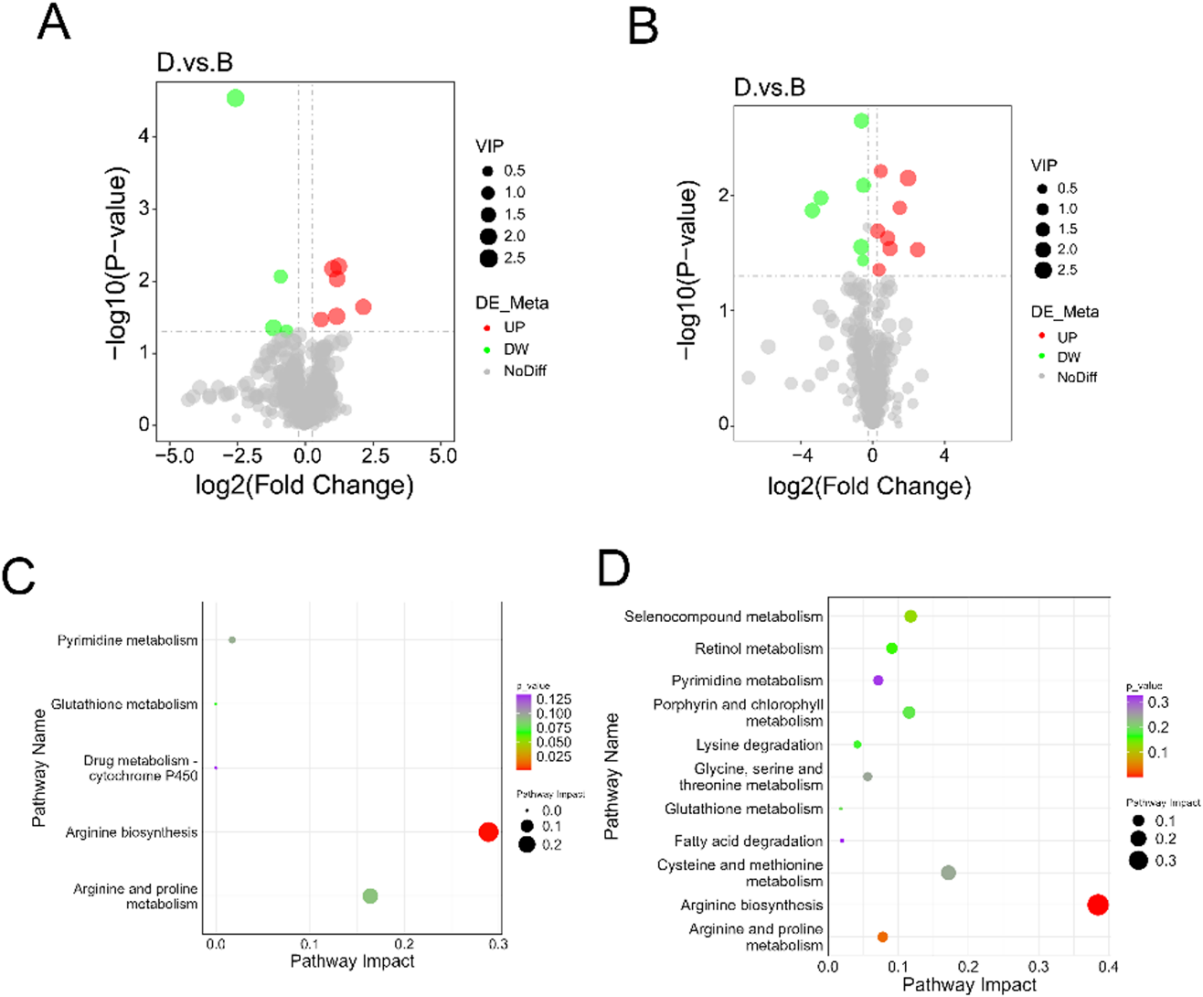
Analysis of differential metabolites in the group with hypertension and periodontitis compared to the periodontitis group, and correlation analysis with differential mRNA. (**A**) Volcano plot of differential metabolites in positive ion mode; (**B**) Volcano plot of differential metabolites in negative ion mode; (**C**) KEGG analysis of differential metabolites in negative ion mode; (**D**) Co-enrichment analysis of KEGG pathways for differential metabolites and differential mRNA under anion mode. In the volcano plot, green dots represent downregulated metabolites, red dots indicate upregulated metabolites, and gray dots denote non-differentially expressed metabolites, with the dot size corresponding to the VIP value. D, Periodontitis with hypertension group; B, periodontitis-only group. In the pathway enrichment plot, the dot size represents the pathway impact value, and the color gradient indicates the level of significance.

We compared the differences in metabolites between the periodontal tissue of rats with periodontitis combined with hypertension and that of rats with periodontitis. Metabolomic data assessment involved the use of principal component analysis (PCA) and partial least square discriminant analysis (PLS-DA) (Fig.S5). The analysis revealed a total of 10 differential metabolites in the positive polarity mode, of which 6 were upregulated and 4 were downregulated (Fig. 6A). In the negative polarity mode, 14 differential metabolites were identified, consisting of 8 upregulated and 6 downregulated metabolites (Fig. 6B). KEGG enrichment analysis indicated that the differential metabolites were primarily enriched in the Arginine biosynthesis pathway (Fig. 6C, Table S5).

### Association analysis of differential genes and differential metabolites in periodontitis with hypertension compared to periodontitis-only

By constructing a correlation network diagram, a joint analysis of differentially expressed transcripts and differential metabolites was performed. The results indicated that, in the positive polarity mode, differentially expressed mRNAs such as Cpne1, Zbtb16, Lce1f, Serpina12, and Mfap3l were centrally positioned in the correlation network alongside differential metabolites including Epitestosterone, Cafestol, Dehydroepiandrosterone (DHEA), D-Homocysteine, and 1-Methylhistidine (Fig.S6A). Additionally, differentially expressed lncRNAs such as Myh8, LOC102550026, XLOC_021074, XLOC_020304, Smad3, and Camk1d were also found to be centrally positioned with the same set of differential metabolites (Fig.S6C). Furthermore, differential miRNAs including rno-miR-450b-5p, rno-miR-483-5p, rno-miR-205, rno-miR-143-5p, and rno-miR-199a-3p were similarly located at the core of the correlation network alongside differential metabolites such as Cafestol, D-Homocysteine, Epitestosterone, Dehydroepiandrosterone (DHEA), 1-Methylhistidine, and lithocholic acid 3-sulfate (Fig.S6E).

In the negative polarity mode, differentially expressed mRNAs such as Cpne1, RT1-M2, Serpina12, and Znf750 were centrally positioned in the correlation network alongside differential metabolites including 8-Iso prostaglandin A2, Thromboxane B2, Lauric acid ethyl ester, 3,4-dihydro-2 H,6 H-[1,3]thiazino[2,3-b]quinazolin-6-one, S-(Methyl)Glutathione, and Citrulline (Fig.S6B). Furthermore, differentially expressed lncRNAs such as LOC102550026, Abt1, Myh8, N4bp1, and Smad3 were also found to be centrally located in the network with differential metabolites including Citrulline, 3,4-dihydro-2 H,6 H-[1,3]thiazino[2,3-b]quinazolin-6-one, Lauric acid ethyl ester, S-(Methyl)Glutathione, and Thromboxane B2 (Fig.S6D). Additionally, differential miRNAs such as rno-miR-143-5p, rno-miR-450b-5p, and rno-miR-450a-5p were similarly situated at the core of the correlation network alongside differential metabolites including S-(Methyl) Glutathione, Citrulline, Ornithine, Indole-2-carboxylic acid, CDP, and Lauric acid ethyl ester (Fig.S6F).

Co-enrichment analysis of differentially expressed genes and differentially expressed metabolites revealed that, in the positive polarity mode, there were no significant common enrichment pathways between the differentially expressed genes and metabolites. In the negative polarity mode, the differential metabolite Citrulline and the differential mRNA Arg1 were found to be co-enriched in the Arginine biosynthesis and Biosynthesis of amino acids metabolic pathways. Additionally, the differential metabolite Ornithine was co-enriched with the differential mRNA Arg1 in the Arginine biosynthesis metabolic pathway (Fig. 6D, Table S6).

### Expression validation of key differential genes in periodontitis with hypertension compared to the periodontitis group

**Fig. 7:**
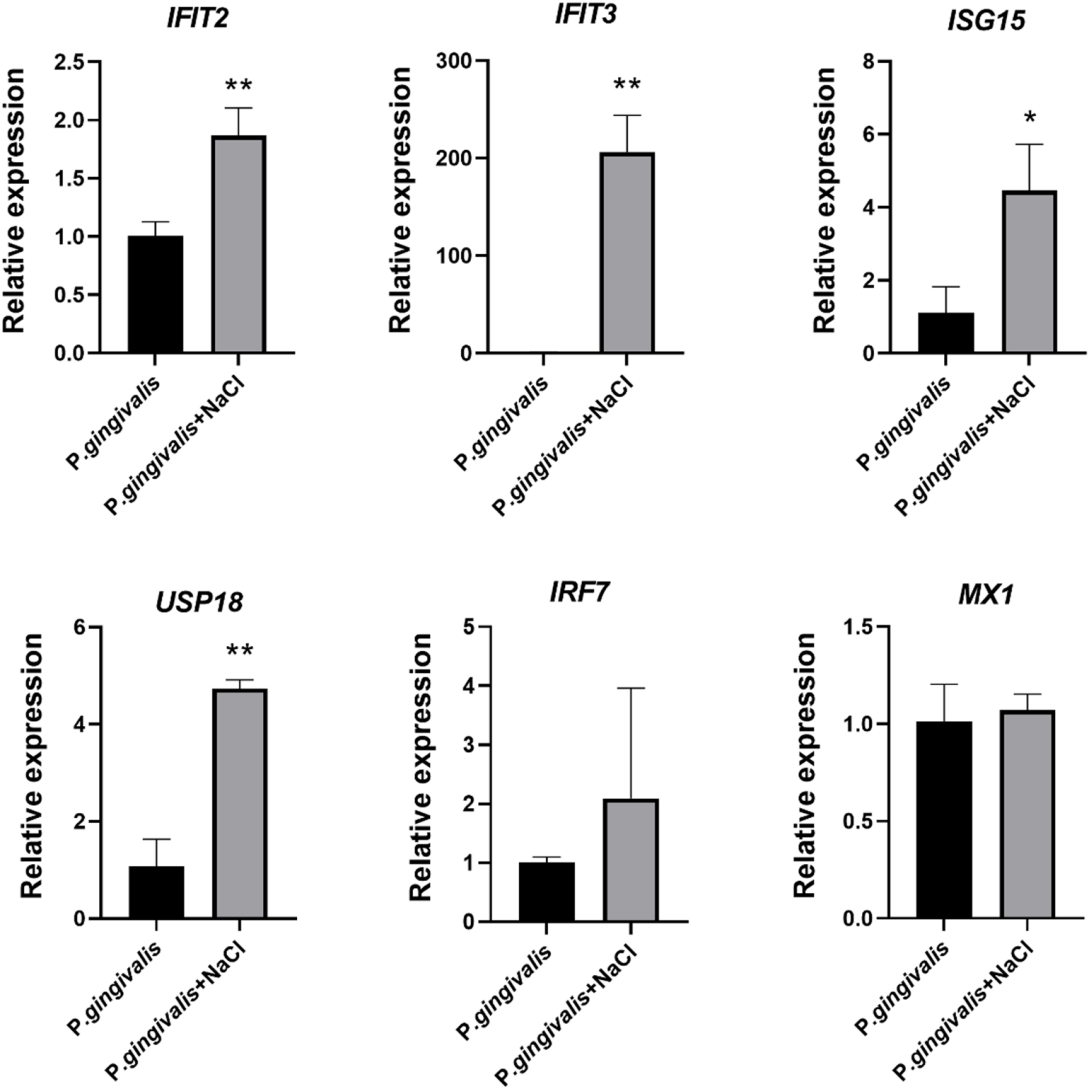
Validation of key differential mRNAs of hypertension combined with periodontitis group compared to periodontitis group. HGFs in hypertension combined with periodontitis group were treated with P.gingivalis (MOI = 100) and NaCl (40mM); **p < 0.01, *p < 0.05.

To further validate the key genes (USP18, ISG15, IFIT3, IRF7, IFIT2, MX1) associated with the development of periodontitis combined with hypertension(Fig. 4D), we cultured human gingival fibroblasts in vitro and stimulated the cells with Porphyromonas gingivalis and sodium chloride (NaCl, 40mM) to mimic the inflammatory response under hypertensive conditions. The mRNA expression of these genes was detected by qRT‑PCR. The results showed that the expression levels of IFIT2, IFIT3, ISG15, and USP18 were significantly higher in cells co‑treated with Porphyromonas gingivalis and NaCl than in cells treated with Porphyromonas gingivalis alone, while the expression of IRF7 and MX1 showed no significant changes (Fig. 7).

### Citrulline attenuates *P. gingivalis* and angiotensin II-induced inflammation in gingival fibroblasts

**Fig. 8:**
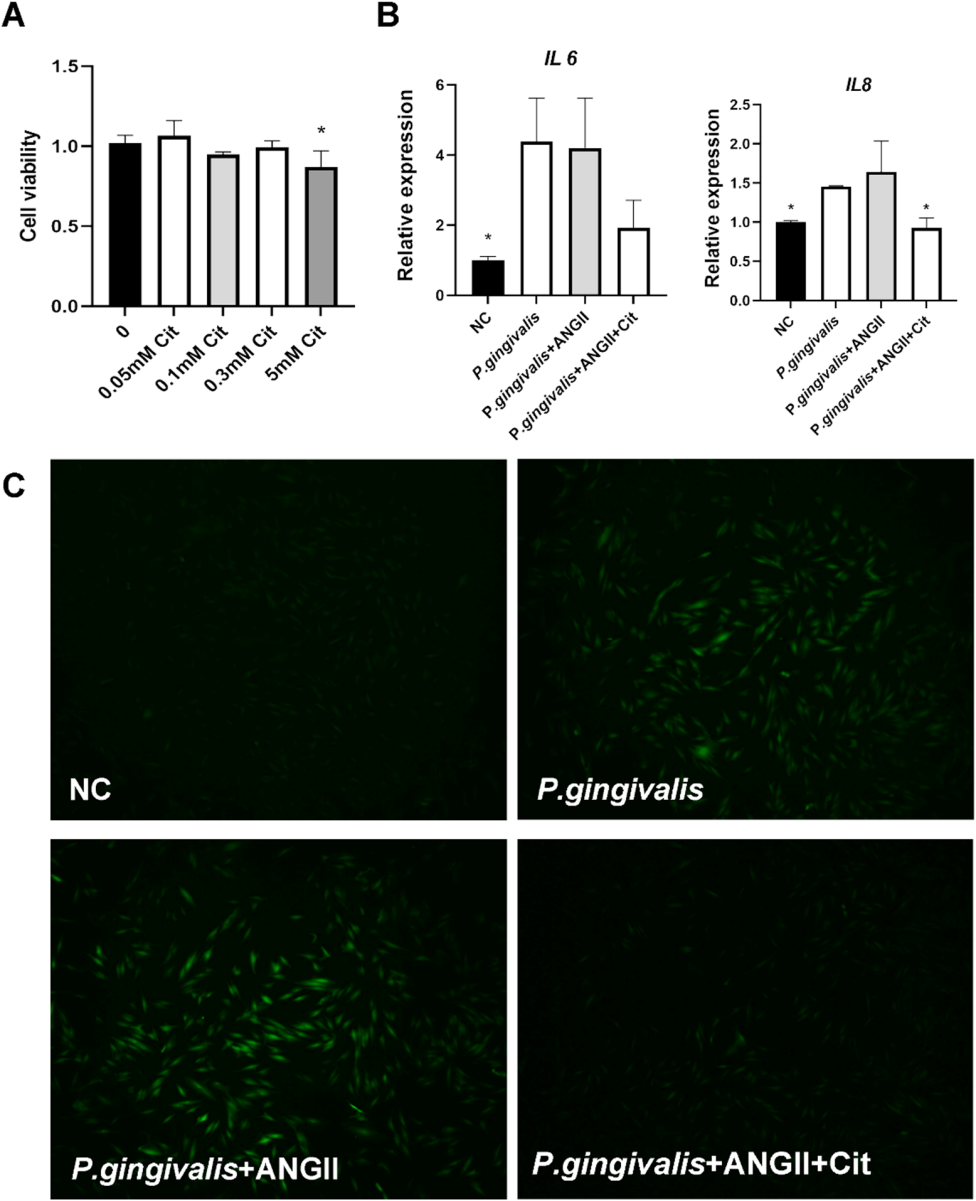
Antioxidant and anti-inflammatory effects of citrulline. (**A**) Viability of HGFs incubated with different concentrations of citrulline; *p < 0.05 compared with 0 mM group; (**B**) Expression levels of IL6 and IL8 in HGFs; *p < 0.05 compared with P.gingivalis + ANG II group; (**C **) intracellular ROS scavenging capacity of citrulline (0.3mM.) using DCFH-DA test; Cit, citrulline.

To validate the omics screening results, we investigated the role of citrulline in periodontitis through in vitro cell experiments. The results indicated that citrulline had no significant effect on the activity of human gingival fibroblasts (Fig. 8A). Angiotensin II, a critical factor in hypertension, enhanced the inflammatory response and promoted reactive oxygen species (ROS) production in gingival fibroblasts induced by P. gingivalis (Fig. 8C). The addition of citrulline reduced ROS generation in gingival fibroblasts (Fig. 8C) and markedly downregulated the expression of the inflammatory cytokine IL8 (Fig. 8B).

## Discussion

In recent years, numerous studies have indicated an independent reciprocal association between hypertension and periodontitis, with hypertension increasing the risk and severity of periodontitis^16,18^. The overall prevalence of periodontitis is higher among hypertensive patients compared to healthy individuals, and the average periodontal indices in the hypertensive group are greater than those in the healthy group. Elevated levels of CRP in the gingival crevicular fluid of hypertensive patients may exacerbate the inflammatory response and alveolar bone loss associated with periodontitis^19^. Additionally, hypertensive patients exhibit a lower rate of recovery from periodontal disease after treatment, suggesting that hypertension may hinder the effectiveness of periodontal therapies^17^. Both hypertension and periodontitis may lead to endothelial dysfunction, which can reduce periodontal vascularization and subsequently impair periodontal health^14^. Spontaneously hypertensive rats show a significant increase in ligature-induced periodontitis and bone loss compared to normotensive rats, indicating that hypertension may exacerbate the progression of periodontitis by affecting blood flow and pressure within periodontal tissues^20^. However, the mechanisms by which hypertension influences periodontitis remain unclear. This study establishes animal models of hypertensive rats and rats with concomitant periodontitis and hypertension, and employs transcriptomic and metabolomic analyses to identify a series of genes and metabolites associated with the occurrence of periodontitis and hypertension. Furthermore, the study analyzes the changes in the transcriptome and metabolites during the development of periodontitis in the context of hypertension, along with the relationship between changes in gene expression and metabolite alterations.

Through the analysis of differentially expressed genes in rat models of periodontitis combined with hypertension compared to healthy rats, genes such as Actn3, Actn2, Tpm1, and Tpm2 were identified as central hubs within the differential gene network, suggesting their potential regulatory roles in the pathogenesis of periodontitis comorbid with hypertension. Among these, Actn2 and Actn3 are primarily associated with muscle function^21,22^. However, emerging evidence also indicates that ACTN3 is involved in muscle inflammatory responses^21,23,24^. In contrast, ACTN2 has been shown to influence blood pressure through its role in cardiac function^25,26^.Additionally, Tpm1 and Tpm2, which encode actin-binding proteins, play critical roles in cytoskeletal regulation. These proteins may contribute to the development of hypertension by modulating actin depolymerization and cytoskeletal dynamics^27–29^.

The changes in metabolites are closely related to the occurrence and progression of periodontitis and can serve as biomarkers or therapeutic targets for periodontitis, aiding in early diagnosis and treatment. In this study, differential metabolites between periodontitis with hypertension rats and healthy rats were compared, and a combined analysis with differentially expressed genes was conducted to identify a series of metabolites and their associated genes related to periodontitis. The results showed that the differential metabolites were mainly enriched in metabolic pathways such as Phenylalanine, tyrosine and tryptophan biosynthesis, Phenylalanine metabolism, and Purine metabolism. Phenylalanine is a precursor of tyrosine and tryptophan, and its metabolic disorder can lead to severe health issues. Elevated levels of phenylalanine are associated with various inflammatory conditions, including acute respiratory distress syndrome (ARDS), where high plasma concentrations are linked to increased mortality^30^. This accumulation may be due to reduced activity of phenylalanine hydroxylase, the enzyme responsible for converting phenylalanine to tyrosine, leading to an imbalance that exacerbates inflammatory responses^30^. Additionally, dysregulation of phenylalanine metabolism leads to oxidative stress, a known factor in the pathogenesis of hypertension^31^.

The association between the purine metabolic pathway and inflammatory hypertension involves complex biochemical processes, particularly the metabolism of uric acid, which is the final product of purine metabolism. Elevated levels of uric acid, or hyperuricemia, are linked to the development and progression of hypertension and related cardiovascular diseases. This relationship is mediated through mechanisms such as oxidative stress, endothelial dysfunction, and inflammation. Uric acid is produced during the breakdown of purines, and xanthine oxidase (XO) plays a crucial role in this process by catalyzing the conversion of hypoxanthine to xanthine and then to uric acid. The elevated levels of XO are associated with increased blood pressure due to the generation of reactive oxygen species (ROS) during purine metabolism, which may lead to endothelial dysfunction and vascular inflammation^32,33,34^. Specifically, the production of superoxide radicals by XO contributes to oxidative stress, a known factor in the pathophysiology of hypertension^33,34^. Furthermore, hyperuricemia has been shown to activate the renin-angiotensin system, exacerbating hypertension by increasing vascular resistance and fluid retention^35,36^.

In this study, L-Tyrosine was significantly downregulated and was found to be involved in metabolic pathways such as Phenylalanine, tyrosine and tryptophan biosynthesis and Phenylalanine metabolism. L-Tyrosine is one of the essential amino acids for collagen synthesis. One of the hallmarks of periodontitis is the destruction of periodontal tissues, including the degradation of collagen. Therefore, L-Tyrosine may play a role in periodontitis by influencing the balance between collagen synthesis and degradation^37^. L-Tyrosine can also be converted into DOPA, which is further synthesized into neurotransmitters such as dopamine and norepinephrine, substances known for their antioxidant properties. Oxidative stress is a significant pathological factor in periodontitis, suggesting that L-Tyrosine may influence the progression of periodontitis through its antioxidant metabolites^38^. Additionally, the differentially expressed gene tyrosine aminotransferase, which also participates in the Phenylalanine, tyrosine and tryptophan biosynthesis and Phenylalanine metabolism pathways, was downregulated. This indicates that during the development of periodontitis, tyrosine aminotransferase may exert a protective effect on the periodontium by regulating L-Tyrosine metabolism, and the reduction of L-Tyrosine could potentially exacerbate the severity of periodontitis.

KEGG and PPI analyses revealed that the expression of interferon-related genes, including Irf7, Isg15, Ifit2, Ifit3, and Mx1, was significantly upregulated in the periodontitis with hypertension group compared to the periodontitis-only group. Irf7 promotes the differentiation of Th1/Th17 cells, driving the secretion of IFN-γ and IL-17, which exacerbates periodontal inflammation^39^. Mx1, an interferon-inducible GTPase, enhances inflammatory responses by activating the AP-1 transcription factor and stimulates type I interferon (IFN-I) production. This further modulates macrophage polarization via the JAK-STAT signaling pathway, thereby influencing periodontal immune regulation^40,41^. Isg15, a ubiquitin-like modifier induced by IFN-I, exhibits elevated expression linked to periodontitis progression. USP18 negatively regulates the IFN-I pathway by deconjugating ISG15 and suppressing IFNAR2 signaling^42,43^. Ifit2 amplifies LPS-induced TNF-α and IL-6 secretion, promoting inflammation, while Ifit3, a marker of M1 macrophages, activates the cGAS/STING pathway and enhances STAT1 expression, driving pro-inflammatory cytokine release^44,45^. In summary, hypertension may synergistically exacerbate the inflammatory response and immune dysregulation in periodontitis by upregulating interferon-related gene expression.

This study revealed that the differential metabolite ornithine, identified between the periodontitis with hypertension group and the periodontitis group, was significantly enriched in the arginine biosynthesis and arginine-proline metabolism pathways. Within these pathways, NOS1 and NOS2 genes catalyze the conversion of arginine to nitric oxide (NO), a critical signaling molecule involved in immune-inflammatory regulation. Previous studies demonstrate that NOS2 gene polymorphisms (rs2779249 and rs2297518) are associated with the incidence of periodontitis in females^46^. Elevated NO levels in periodontal tissues and plasma of periodontitis patients correlate positively with disease severity^47^. Notably, NO exhibits dual regulatory effects by suppressing pro-inflammatory cytokines (e.g., IL-1β, TNF-α) and NLRP3 inflammasome assembly^48^. In this study, NOS2 was significantly upregulated in the periodontitis with hypertension group compared to healthy controls, suggesting its potential role in promoting periodontitis progression through arginine biosynthesis and arginine-proline metabolism pathways.

The Arg1 gene, co-enriched with citrulline and ornithine in arginine biosynthesis pathway, participates in inflammatory regulation. Arginine metabolism involves two competitive pathways: the arginase pathway (producing ornithine and urea) and the nitric oxide synthase (NOS)-mediated NO production pathway. As an M2 macrophage marker, Arg1 upregulation may inhibit NO generation by substrate competition while promoting anti-inflammatory polyamine synthesis, thereby suppressing M1 macrophage polarization^49^. Intriguingly, although Arg1 expression was significantly elevated in the periodontitis with hypertension group compared to the periodontitis group, ornithine and citrulline levels decreased, indicating Arg1-mediated dysregulation of arginine metabolism might synergize with hypertension to exacerbate periodontitis. Furthermore, ornithine cycle metabolites (e.g., N-acetylornithine) correlate with cytokine storm severity^50^. Clinical evidence shows that Arg1 overexpression in gingival tissues associates with periodontitis severity and Porphyromonas gingivalis infection^51^, reinforcing its pivotal role in inflammation-repair balance. Collectively, hypertension-comorbid periodontitis may reprogram arginine metabolism to influence macrophage polarization and NO homeostasis, thereby modulating inflammatory progression. The synergistic interaction between Arg1 and NOS2 likely constitutes a critical node linking hypertension and periodontitis pathogenesis, providing a theoretical basis for targeted metabolic interventions.

In this study, key genes including USP18, ISG15, IFIT3, IRF7, IFIT2, and MX1 were identified through high‑throughput sequencing combined with analysis using the Cytohubba plugin of CytoScape. The results demonstrated that these genes are highly expressed in periodontal tissues affected by inflammation combined with hypertension. Quantitative reverse‑transcription polymerase chain reaction (qRT‑PCR) further confirmed the elevated expression of Usp18, Isg15, Ifit3, and Ifit2 in gingival fibroblasts treated with Porphyromonas gingivalis and sodium chloride. These findings suggest that these genes play important roles in the pathological context of periodontitis comorbid with hypertension. Current evidence indicates that genes such as USP18, IFIT2, IFIT3, and ISG15 are key members of the type I interferon‑stimulated gene (ISG) family and exert critical functions in innate immune responses and inflammatory regulation. For example, IFIT2 is essential for antiviral defense by restricting the spread of neurotropic murine beta‑coronavirus (RSA59) infection and limiting associated myelin pathology in the spinal cord white matter^52^. IFIT2 also confers protection against vesicular stomatitis virus (VSV) infection^53^. Moreover, IFIT2 limits autoimmune inflammation by regulating the activation and metabolic activity of myeloid cells^54^. In ulcerative colitis, downregulation of IFIT3 alleviates the inflammatory response through selective modulation of macrophage M1 polarization and the STAT1/2 signaling pathway^55^. ISG15, one of the most abundantly induced genes by type I interferon, can act as a cytokine that binds to cell‑surface receptors to regulate immune‑cell function and inflammatory responses^56^. During SARS‑CoV‑2 infection, SARS‑CoV‑2 induces macrophages to secrete free ISG15, thereby exacerbating virus‑triggered inflammation^57,58^. USP18 plays a significant regulatory role in various inflammatory diseases; it mitigates lipopolysaccharide (LPS)‑induced oxidative stress and inflammation^59^, and negatively regulates LPS‑induced sepsis by targeting the activity of transforming growth factor‑β‑activated kinase 1 (TAK1)^60^.

To further validate the role of citrulline in periodontitis, this study conducted in vitro cell experiments. The results demonstrated that citrulline alleviated the inflammatory response in gingival fibroblasts induced by P.gingivalis and Ang II. Multiple studies have indicated that citrulline can significantly suppress the production of pro-inflammatory cytokines in synovial cells, macrophages, and COVID-19 patients^61–63^, which is consistent with the findings of the present study. Furthermore, citrulline is recognized as a precursor to arginine, which can be converted to arginine in vivo to subsequently generate nitric oxide (NO)^64^. NO plays a crucial role in regulating vascular tone, platelet aggregation, and immune responses^65^. Through this pathway, citrulline can influence vascular function and immune reactivity. One study revealed that citrulline mitigates inflammation and jejunal damage in intestinal ischemia-reperfusion injury by inactivating neuronal nitric oxide synthase (nNOS) and nuclear factor-kappa B (NF-κB)^66^. Notably, our omics analysis revealed that in the periodontal tissues of subjects with hypertension and periodontitis, NOS1 expression was significantly downregulated, while NOS2 expression was significantly upregulated (Figs. 1A and 4A). This suggests that citrulline might also modulate the pathogenesis of hypertension-associated periodontitis via NOS2.

## Materials and methods

### Animals

Six-week-old male Sprague-Dawley (SD) rats, weighing 180–220 g, were purchased from the Experimental Animal Center of Guangxi Medical University, with the license number SCXK Gui 2020-0003. All animal care and experimental methods were performed in accordance with the relevant guidelines and regulations approved by the Animal Ethics Committee of Guangxi Medical University (Approval Number: 202206060), and they adhered to the ARRIVE guidelines. The SD rats were maintained between 22 and 24℃ in standard ventilated cages holding 3 rats per cage and water ad libitum. A total of nine animals were randomly divided into three different groups, periodontitis group (P), periodontitis combined with hypertension group (PH), negative control group (NC). The rats in the PH groups were fed a high-salt diet (containing 8% NaCl) for 7 weeks, while the rats in the NC and P groups were given standard maintenance feed, with all groups allowed free access to food and water. In a quiet state, the systolic blood pressure, diastolic blood pressure, and heart rate of the rats were recorded at the same time each week using the Softron non-invasive blood pressure monitor for mice and rats (BP-2010 A). The average value was calculated after three consecutive measurements. After 7 weeks, the mandibular first molars in the P and PH groups were ligated with sterile 3 − 0 silk sutures to establish a periodontitis model. Four weeks after ligation, rats were euthanized by CO2 in a 43-L chamber at a flow rate of 13 L/min (Patterson Scientific anesthesia machine, USA). The gingival tissues of the mandibular first molars were collected for sequencing analysis.

### RNA extraction and sequencing

Each sample was split into two equal parts: one-half was used for metabolomic analysis and the other half for transcriptomic analysis. sequencing libraries were constructed with TruSeq RNA Sample Preparation Kits v2 (Illumina) or NEB Next^®^ Multiplex Small RNA Library Prep Set for Illumina^®^ (NEB E7300L) for mRNA/lncRNA or miRNA sequencing respectively. library quality was assessed on the Agilent 5400 system and quantified by QPCR. The Qualified libraries were pooled and sequenced on Illumina platforms with PE150 strategy in Novogene Bioinformatics Technology Co., Ltd (Beijing, China), according to effective library concentration and data amount required.

### Differential gene screening and functional enrichment analysis

Differential mRNA and lncRNA expression was determined using edgeR according to the filter criteria (log2|fold change| >=0.0, padj < 0.05), and differential miRNA expression analysis was performed using the DESeq R package (1.8.3) according to the filter criteria (|log2FoldChange|>=0, pvalue < = 0.05). Enrichment analyses, including KEGG (https://www.kegg.jp/kegg/)^67–69^and gene ontology (GO) were conducted using clusterprofiler package for R software. lncRNA–miRNA–mRNA regulatory network was constructed according the whole transcriptome sequencing (WTS).

### Metabolite extraction

After transferring 100 mg of tissue sample ground in liquid nitrogen into an EP tube, add 500 µL of an 80% aqueous methanol solution. Vigorously vortex the mixture and let it sit in an ice bath for 5 min to enhance extraction. Subsequently, centrifuge the tube at 15000 g and 4℃ for 20 min to separate the solid particles from the liquid extract. Carefully collect a specific volume of the supernatant and dilute it with high-quality water to achieve a methanol content of 53%. Once again, centrifuge the diluted sample at 15000 g and 4℃ for 20 min to remove any remaining debris. Finally, collect the clarified supernatant for further analysis using LC-MS.

### Metabolite detection

UHPLC-MS/MS analyses were conducted at Novogene Co., Ltd. (Beijing, China) using a Vanquish UHPLC system (ThermoFisher, Germany) coupled with either an Orbitrap Q ExactiveTM HF or Orbitrap Q ExactiveTM HF-X mass spectrometer (Thermo Fisher, Germany). Samples were injected onto a Hypersil Gold column (100 × 2.1 mm, 1.9 μm) and separated using a 12-minute linear gradient at a flow rate of 0.2 mL/min. For the positive polarity mode, eluent A (0.1% FA in Water) and eluent B (Methanol) were used, while for the negative polarity mode, the same eluents were employed. The solvent gradient was set as follows: starting with 2% B for 1.5 min, followed by a linear increase from 2% to 85% B over 3 min, then from 85% to 100% B over 10 min. Subsequently, the gradient was reversed from 100% to 2% B over 10.1 min and held at 2% B for an additional 12 min.

The Q ExactiveTM HF mass spectrometer was operated in both positive and negative polarity modes with a spray voltage of 3.5 kV. The capillary temperature was set at 320 °C, the sheath gas flow rate at 35 psi, and the auxiliary gas flow rate at 10 L/min. The S-lens RF level was set to 60, and the auxiliary gas heater temperature was maintained at 350 °C.

### Metabolome data processing and analysis

The obtained raw data of the metabolome were processed using Compound Discoverer 3.3 software in combination with mzCloud (https://www.mzcloud.org/), mzVault, and Masslist databases for metabolite identification and relative quantification. The identified metabolites were annotated using the KEGG database (https://www.genome.jp/kegg/pathway.html), HMDB database (https://hmdb.ca/metabolites), and LIPIDMaps database (http://www.lipidmaps.org/). The metabolomics data processing software, metaX, was used to transform the data, followed by principal component analysis (PCA) and partial least squares discriminant analysis (PLS-DA) to obtain the variable importance in projection (VIP) values for each metabolite.

In the univariate analysis, t-tests were performed to calculate the statistical significance (P-value) and fold change (FC) values of each metabolite between the two groups. The criteria for differential metabolite selection were set as VIP > 1, P-value < 0.05, and FC ≥ 2 or FC ≤ 0.5. The online tool MetaboAnalyst 6.0 was used to study the functions and metabolic pathways of the identified metabolites.

### Transcriptome and metabolome associative analysis

Pearson correlation coefficient was employed to assess the relationship between the transcriptome and metabolome, aiming to evaluate the degree of association between differentially expressed genes identified in the transcriptome analysis and differentially abundant metabolites detected in the metabolome analysis. The correlation coefficient reflects the strength and direction of the relationship between the two datasets. A negative correlation coefficient indicates an inverse relationship, while a positive correlation coefficient signifies a direct relationship. The screening criteria were defined as an absolute value of Pearson correlation coefficient > 0.8 with a significance level of *P* < 0.05. The analysis of co-enriched KEGG pathways between differentially expressed genes and differentially expressed metabolites was conducted using the online tool MetaboAnalyst 6.0.

### Isolation of human gingival fibroblasts

Human gingival fibroblasts (HGFs) were obtained from patients aged 15 to 25 years undergoing crown lengthening surgery at the Department of Periodontics and Oral Medicine, Hospital of Stomatology, Guangxi Medical University. After washing the tissues with PBS, they were cut into pieces (1 mm×1 mm) and digested with type I collagenase (Sigma, St. Louis, MO, USA) for 2 h at 37℃. The dispersed tissues were placed on the bottom l of T25 flasks and maintained in alpha MEM supplemented with 20% fetal bovine serum in a humidified incubator (37 °C, 5% CO2).

### Cellular viability assay

HGFs were seeded in 96-well plates with a density of 5000 cells per well and cultured for 12 h. Different concentrations of citrulline (Solarbio, Beijing, China) were added to the wells. After 24 h, fresh medium containing 10% CCK-8 solution was added and the cells were incubated for another 1 h. Absorbance was measured at 450 nm by a microplate reader.

### RNA isolation and quantitative RT-PCR

Total RNA was extracted from the HGFs s using FreeZol Reagent (Vazyme, Nanjing, China) and reverse-transcribed into first-strand cDNA (Takara, Kusatsu, Shiga, Japan) according to the manufacturer’s instructions. The resulting cDNA was used as a template for quantitative RT-PCR with RealStar Fast qPCR Mix (GenStart, Beijing, China). Reactions were run on the QuantStudio 5 PCR System (Applied Biosystems). The relative mRNA expressions were calculated as fold changes normalized to GAPDH expression. The primers were listed in the table S7.

### Intracellular ROS scavenging activities

HGFs were plated in 96-well plates with a density of 5000 cells per well and cultured for 12 h. The cells were preincubated with citrulline (0.3mM) for 1 h before stimulation with ANG II (0.04mM; Solarbio, Beijing, China) for 12 h. The combined treatment group, cells were further stimulated with Porphyromonas gingivalis (MOI = 200) for another 12 h. After washing with PBS, the cells were incubated with fresh medium containing DCFH-DA for 20 min at 37 °C in dark. Fluorescence images were acquired with an Olympus imaging system.

## Conclusions

By establishing rat models of periodontitis and periodontitis combined with hypertension, along with joint analysis of transcriptomics and metabolomics, it was found that hypertension may promote the progression of periodontitis by altering interferon-induced genes and arginine metabolism-related pathways. Citrulline represents a promising candidate for the treatment of periodontitis associated with hypertension. The specific mechanisms underlying this process require further experimental investigation for validation.

## Supplementary Information

Below is the link to the electronic supplementary material.

Supplementary Material 1

Supplementary Material 2

Supplementary Material 3

Supplementary Material 4

Supplementary Material 5

Supplementary Material 6

Supplementary Material 7

Supplementary Material 8

## Data Availability

The original contributions presented in this study are included in the article/supplementary material. High-throughput sequencing data supporting the findings of this study have been uploaded to Gene Expression Omnibus (GEO) with accession numbers GSE295190 (token number: ybmjyikklhyrtkv) and GSE295193 (token number: efifaiwktluntef).
