# Supplementary figures and images for: Integrated transcriptomic and metabolomic analysis to elucidate key genes and signaling pathways involved in the promotion of periodontitis by hypertension
Source: Sci Rep. 2026 Apr 13;16:17103. doi: 10.1038/s41598-026-48279-8 (PMC13230694; doi:10.1038/s41598-026-48279-8)

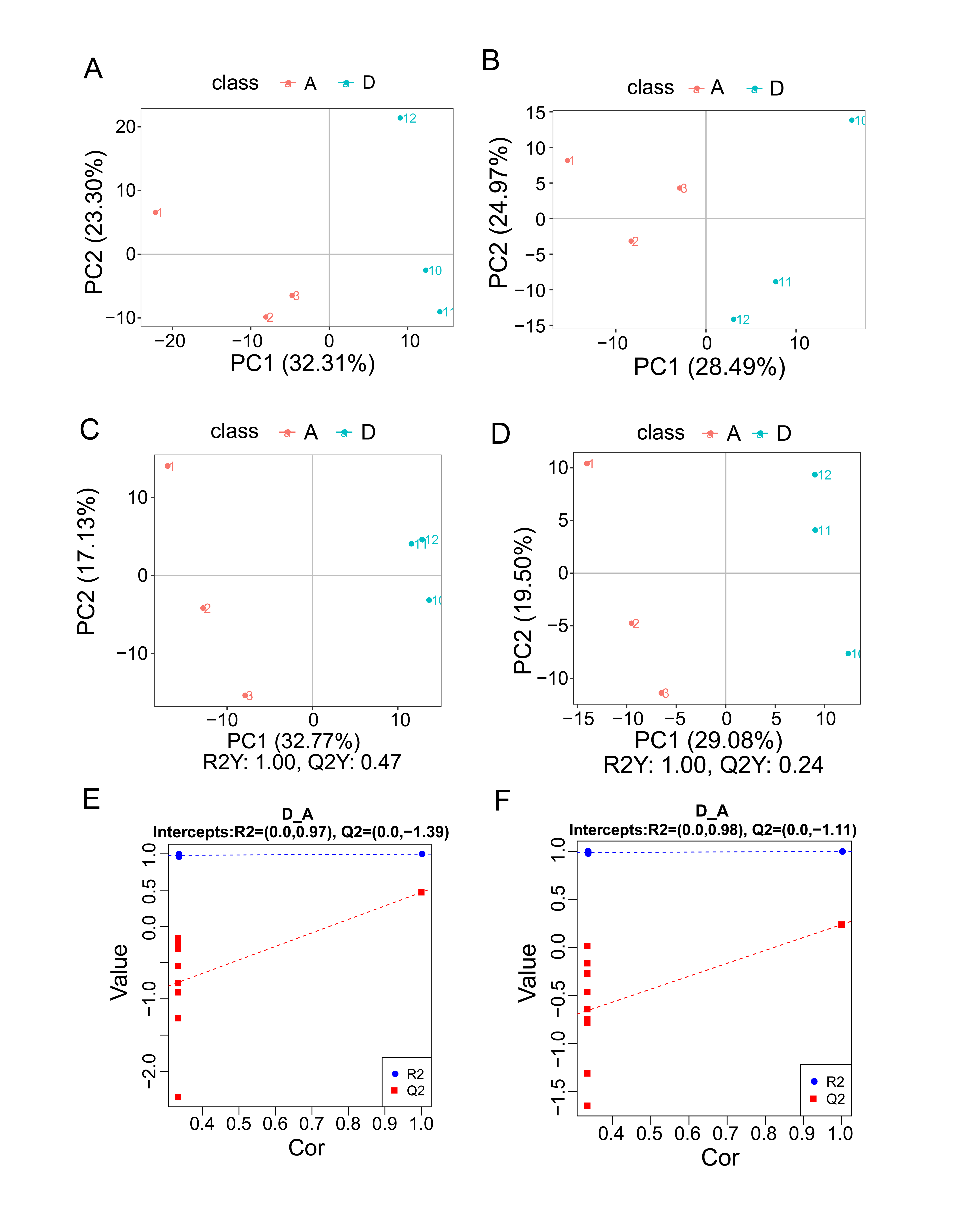

Supplement: Supplementary file 1 — Supplementary Material 1 [file 41598_2026_48279_MOESM1_ESM.tif]

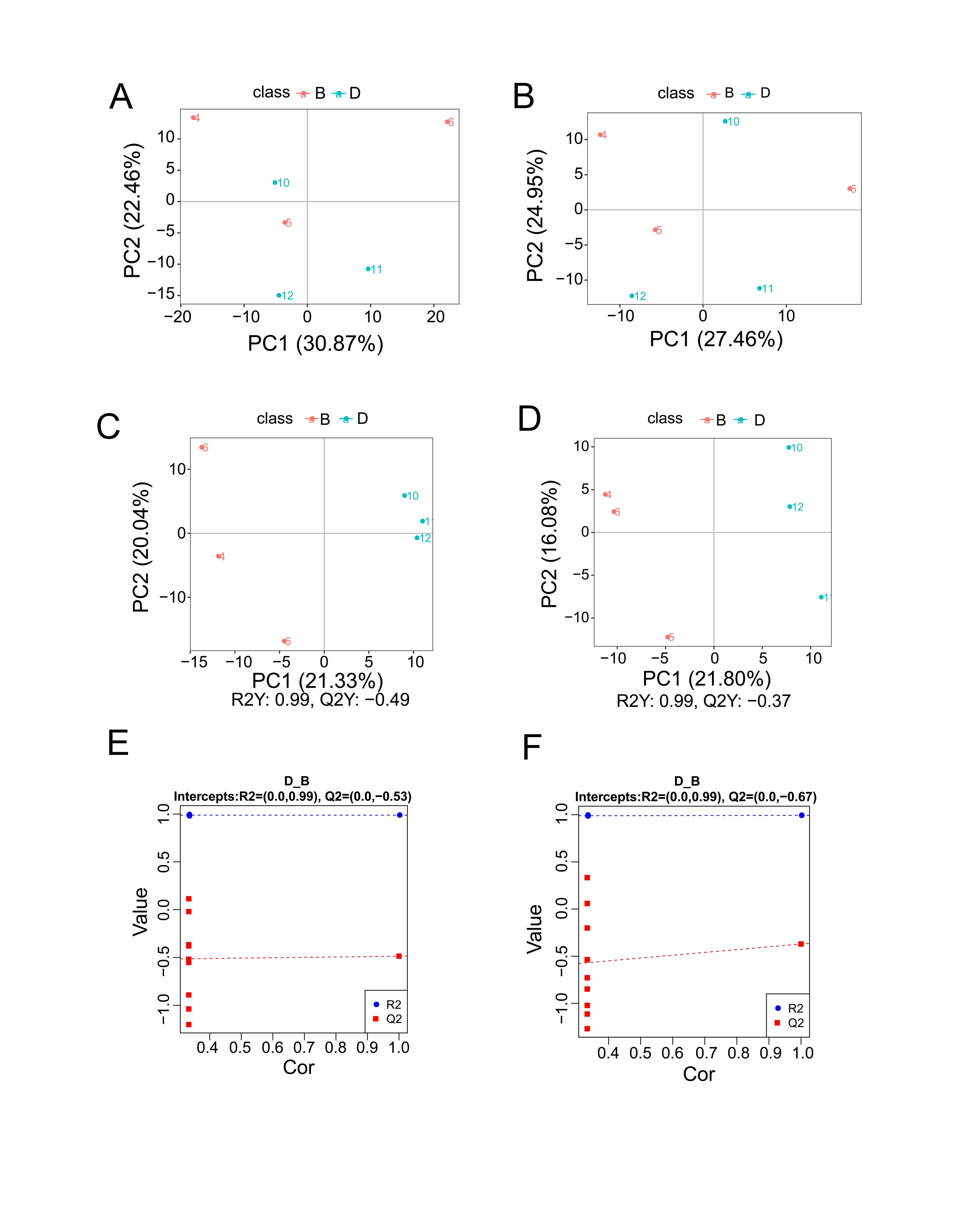

Supplement: Supplementary file 2 — Supplementary Material 2 [file 41598_2026_48279_MOESM2_ESM.tif]

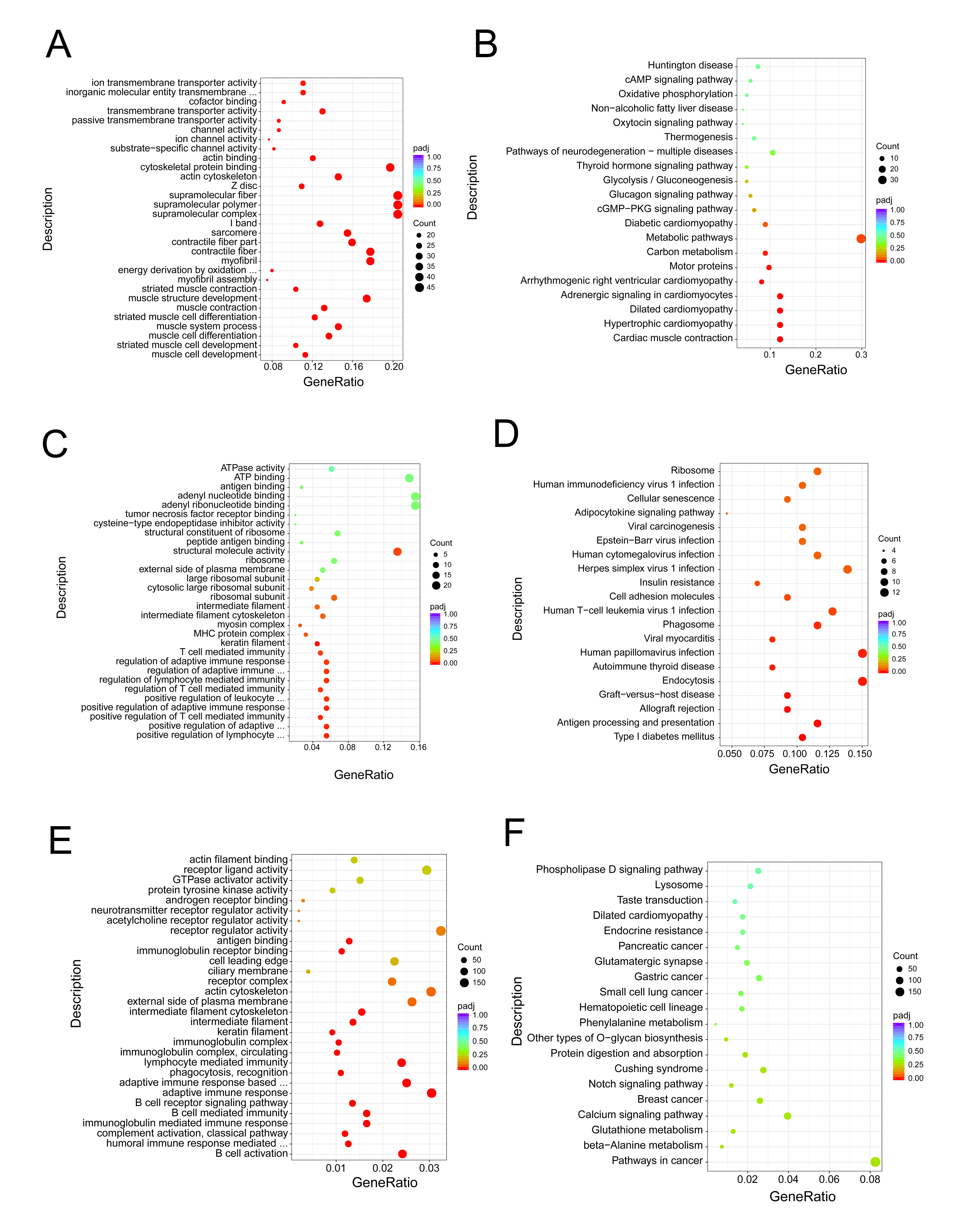

Supplement: Supplementary file 3 — Supplementary Material 3 [file 41598_2026_48279_MOESM3_ESM.tif]

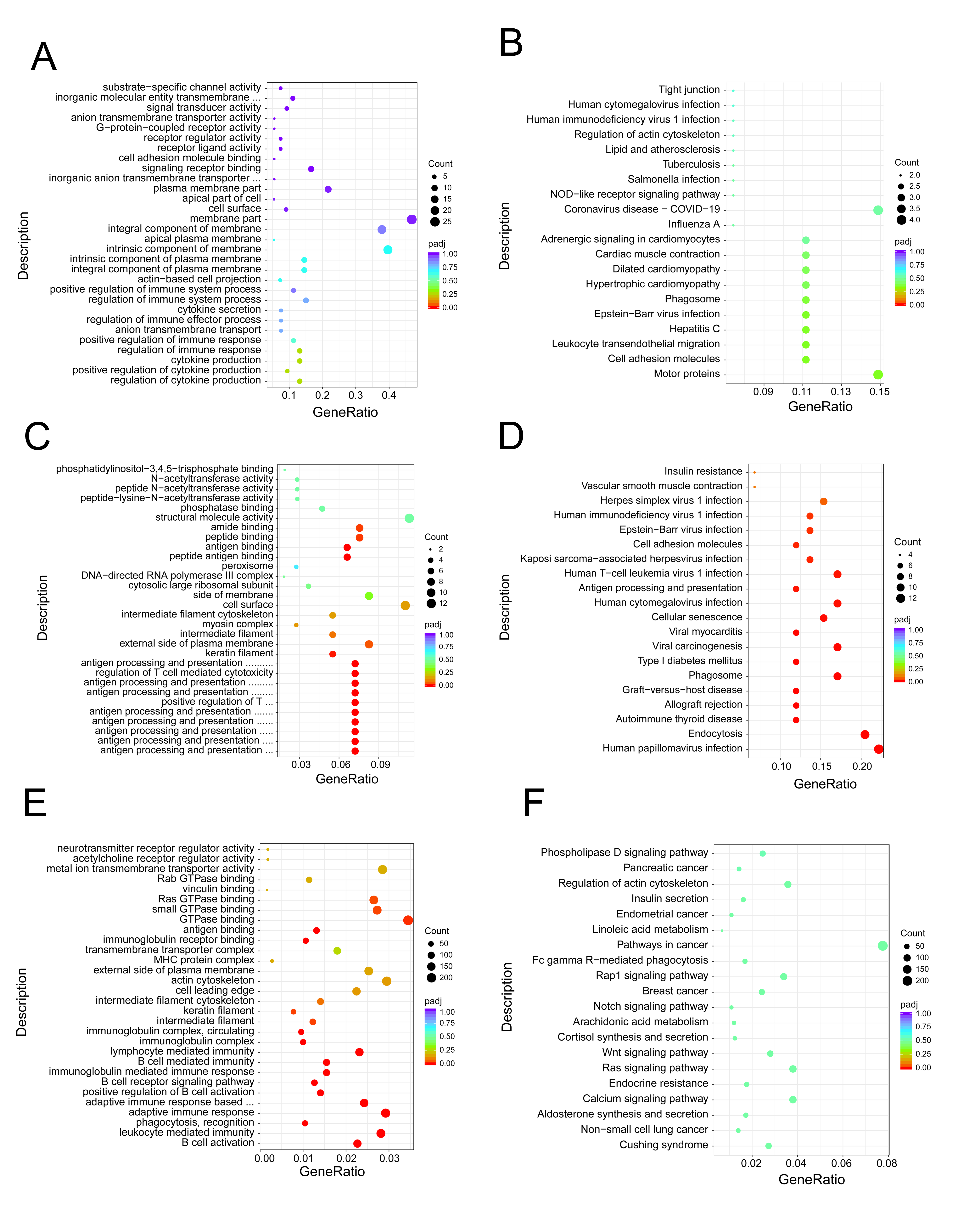

Supplement: Supplementary file 4 — Supplementary Material 4 [file 41598_2026_48279_MOESM4_ESM.tif]

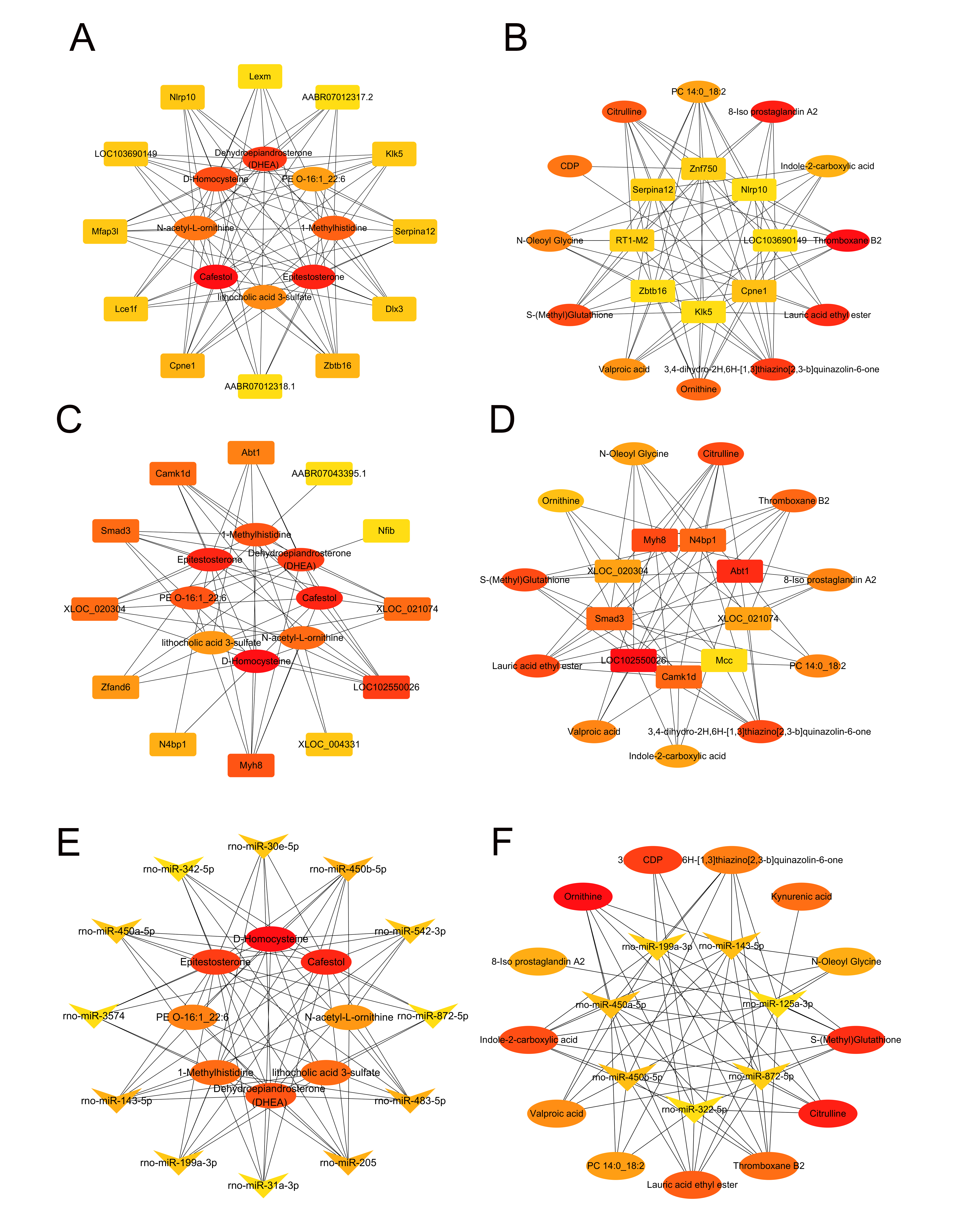

Supplement: Supplementary file 5 — Supplementary Material 5 [file 41598_2026_48279_MOESM5_ESM.tif]

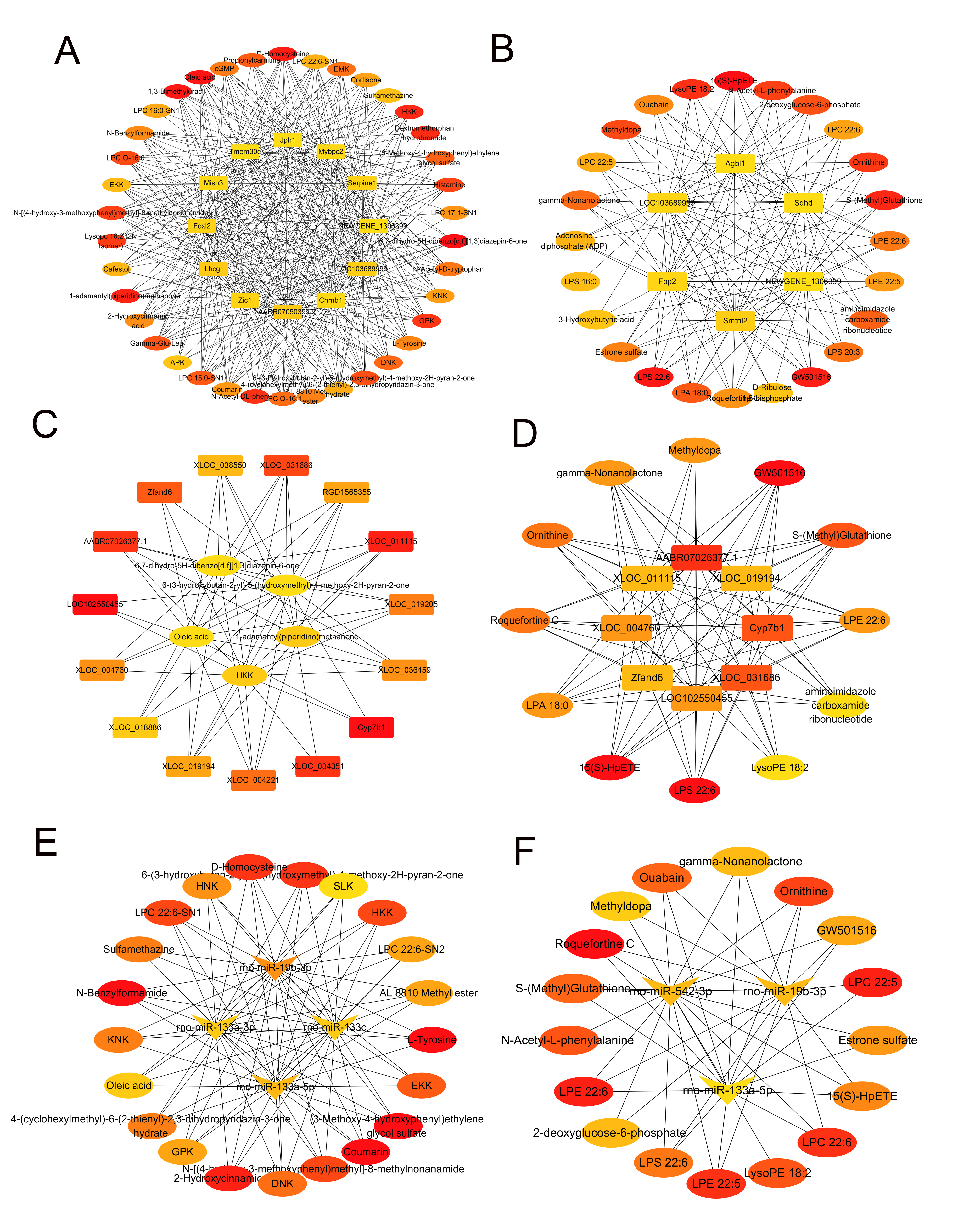

Supplement: Supplementary file 6 — Supplementary Material 6 [file 41598_2026_48279_MOESM6_ESM.tif]
