# Supplementary material for: Integrated transcriptomic and metabolomic analysis to elucidate key genes and signaling pathways involved in the promotion of periodontitis by hypertension
Source: Sci Rep. 2026 Apr 13;16:17103. doi: 10.1038/s41598-026-48279-8 (PMC13230694; doi:10.1038/s41598-026-48279-8)
Supplement: Supplementary file 7 — Supplementary Material 7 [file 41598_2026_48279_MOESM7_ESM.docx]

**Supplementary figure legends**

**Fig.S1.** GO and KEGG enrichment analysis of co-expressed genes and co-located genes in differentially expressed lncRNAs, as well as differentially expressed miRNA target genes, in the periodontitis combined with hypertension group compared to the control group. (A) GO enrichment analysis of co-expressed genes of differential lncRNAs; (B) KEGG enrichment analysis of co-expressed genes of differential lncRNAs; (C) GO enrichment analysis of co-localized genes of differential lncRNAs; (D) KEGG enrichment analysis of co-localized genes of differential lncRNAs; (E) GO enrichment analysis of target genes of differential miRNAs; (F) KEGG analysis of target genes of differential miRNAs. The dot size represents the number of enriched genes, the color gradient indicates the level of significance.

**Fig.S2.** Principal Component Analysis (PCA) and Partial Least Squares Discriminant Analysis (PLS-DA) of the metabolomic data in the hypertension combined with periodontitis group compared to the control group. (A) PCA analysis in positive ion mode; (B) PCA analysis in negative ion mode; (C) PLS-DA analysis in positive ion mode; (D) PLS-DA analysis in negative ion mode; (E) Model validation by permutation test for PLS-DA in positive ion mode; (F) Model validation by permutation test for PLS-DA in negative ion mode. A，control group; D, periodontitis with hypertension group

**Fig.S3.** Correlation analysis between differential lncRNAs, differential miRNAs, and differential metabolites in the hypertension combined with periodontitis group compared to the control group. (A) Correlation analysis between differential mRNAs and differential metabolites in positive ion mode; (B) Correlation analysis between differential mRNAs and differential metabolites in negative ion mode; (C) Correlation analysis between differential lncRNAs and differential metabolites in positive ion mode; (D) Correlation analysis between differential lncRNAs and differential metabolites in negative ion mode; (E) Correlation analysis between differential miRNAs and differential metabolites in positive ion mode; (F) Correlation analysis between differential miRNAs and differential metabolites in negative ion mode. Rounded rectangles represent mRNAs or lnRNAs, V-shapes represent mirnas, and ellipses represent metabolites. The color intensity corresponds to the likelihood of being hub nodes.

**Fig.S4.** GO and KEGG enrichment analysis of co-expressed genes and co-located genes in differentially expressed lncRNAs, as well as differentially expressed miRNA target genes, in the hypertension combined with periodontitis group compared to the periodontitis group. (A) GO enrichment analysis of co-expressed genes of differential lncRNAs; (B) KEGG enrichment analysis of co-expressed genes of differential lncRNAs; (C) GO enrichment analysis of co-localized genes of differential lncRNAs; (D) KEGG enrichment analysis of co-localized genes of differential lncRNAs; (E) GO enrichment analysis of target genes of differential miRNAs; (F) KEGG analysis of target genes of differential miRNAs. The dot size represents the number of enriched genes, the color gradient indicates the level of significance.

**Fig.S5.** Principal Component Analysis (PCA) and Partial Least Squares Discriminant Analysis (PLS-DA) of the metabolomic data in the periodontitis combined with hypertension group compared to the periodontitis group. (A) PCA analysis in positive ion mode; (B) PCA analysis in negative ion mode; (C) PLS-DA analysis in positive ion mode; (D) PLS-DA analysis in negative ion mode; (E) Model validation by permutation test for PLS-DA in positive ion mode; (F) Model validation by permutation test for PLS-DA in negative ion mode. B, periodontitis-only group; D, periodontitis combined with hypertension group.

**Fig.S6.** Correlation analysis between differential lncRNAs, differential miRNAs, and differential metabolites in the hypertension combined with periodontitis group compared to the periodontitis group. (A) Correlation analysis between differential mRNAs and differential metabolites in positive ion mode; (B) Correlation analysis between differential mRNAs and differential metabolites in negative ion mode; (C) Correlation analysis between differential lncRNAs and differential metabolites in positive ion mode; (D) Correlation analysis between differential lncRNAs and differential metabolites in negative ion mode; (E) Correlation analysis between differential miRNAs and differential metabolites in positive ion mode; (F) Correlation analysis between differential miRNAs and differential metabolites in negative ion mode. Rounded rectangles represent mRNAs or lnRNAs, V-shapes represent mirnas, and ellipses represent metabolites. The color intensity corresponds to the likelihood of being hub nodes.
