# Supplementary material for: Integrated transcriptomic and metabolomic analysis to elucidate key genes and signaling pathways involved in the promotion of periodontitis by hypertension
Source: Sci Rep. 2026 Apr 13;16:17103. doi: 10.1038/s41598-026-48279-8 (PMC13230694; doi:10.1038/s41598-026-48279-8)
Supplement: Supplementary file 8 — Supplementary Material 8 [file 41598_2026_48279_MOESM8_ESM.docx]

**Table S1. KEGG Enrichment Analysis of Differentially Expressed Genes in Periodontitis with Hypertension Compared to the Healthy Group (Top 10)**

| KEGG ID | Description | Gene Ratio | P-value | Gene Name |
| --- | --- | --- | --- | --- |
| rno04260 | Cardiac muscle contraction | 20/287 | 2.64E-08 | Hrc/Cacna1s/XLOC_004015/Atp1a2/AABR07005775.1/Trdn/Tpm1/Atp1b1/Tpm2/Cox8b/Cox6a2/Actc1/Myh7/Cacna2d1/Myh6/Myl3/Atp1b4/Cacng6/Cacng1/Cacnb1 |
| rno05410 | Hypertrophic cardiomyopathy | 20/287 | 3.11E-08 | AABR07052585.2/XLOC_023727/Cacna1s/XLOC_004015/AABR07052585.1/AABR07005775.1/Tpm1/Des/Tpm2/Actc1/Sgcg/Myh7/Cacna2d1/Myh6/Myl3/Cacng6/Sgcb/Cacng1/Cacnb1/Prkag3 |
| rno03320 | PPAR signaling pathway | 17/287 | 1.97E-07 | Plin4/Fabp3/Cpt1b/RGD1565355/Aqp7/Fabp4/Sorbs1/Plin1/Ppara/Me3/Acox2/Plin5/Scd/Acsl6/Adipoq/Fabp5/Olr1 |
| rno05414 | Dilated cardiomyopathy | 19/287 | 2.40E-07 | AABR07052585.2/XLOC_023727/Cacna1s/XLOC_004015/AABR07052585.1/AABR07005775.1/Tpm1/Des/Tpm2/Actc1/Sgcg/Myh7/Cacna2d1/Myh6/Myl3/Cacng6/Sgcb/Cacng1/Cacnb1 |
| rno04261 | Adrenergic signaling in cardiomyocytes | 22/287 | 2.81E-06 | Cacna1s/XLOC_004015/Atp1a2/AABR07005775.1/Tpm1/Atp1b1/Tpm2/Scn4b/Actc1/Popdc2/Myh7/Cacna2d1/Myh6/Myl3/Popdc3/Atp1b4/Cacng6/Scn1b/Mapk12/Cacng1/Cacnb1/Plcb4 |
| rno04814 | Motor proteins | 21/287 | 0.000195082 | Tnnc2/Tnnt3/Myh8/Myo18b/Myh1/Myl1/Mylpf/Tpm1/Acta1/Tpm2/Actc1/Myh7/Myh6/Myl3/Kif26b/Dynll1/Actg2/Tnnt1/Dnah11/Myh3/Myh11 |
| rno05412 | Arrhythmogenic right ventricular cardiomyopathy | 13/287 | 0.000328699 | Actn2/Cacna1s/XLOC_004015/AABR07005775.1/Des/Sgcg/Actn3/Cacna2d1/Cacng6/Sgcb/AABR07061178.1/Cacng1/Cacnb1 |
| rno04657 | IL-17 signaling pathway | 14/287 | 0.000385645 | S100a9/S100a8/Mmp13/Cxcl6/Cxcl10/LOC100912585/Defb4/Mmp9/Cxcl3/Ptgs2/Il1b/Ccl11/Ccl12/Mapk12 |
| rno04974 | Protein digestion and absorption | 13/287 | 0.000588216 | Atp1a2/Col8a1/Atp1b1/Col1a1/Col11a1/Col16a1/Col4a2/Col12a1/Col27a1/Col4a1/Col7a1/Atp1b4/Col18a1 |
| rno04922 | Glucagon signaling pathway | 14/287 | 0.000727992 | Pygm/Pgam2/Pfkm/Cpt1b/Acacb/Ldhb/Gys1/Fbp2/Phkg1/Ppara/Ppargc1a/Phka1/Plcb4/Prkag3 |

**Table S2. KEGG Enrichment Analysis of Differential Metabolites in Periodontitis with Hypertension Compared to the Healthy Group**

| **pathway name** | **Total** | **Hits** | **P-value** | **Impact** | **Metabolism** | **Polarity mode** |
| --- | --- | --- | --- | --- | --- | --- |
| Phenylalanine, tyrosine and tryptophan biosynthesis | 4 | 1 | 0.020182 | 0.5 | L-Tyrosine | positive |
| Phenylalanine metabolism | 8 | 1 | 0.040007 | 0 | L-Tyrosine | positive |
| Histidine metabolism | 16 | 1 | 0.078607 | 0.18852 | Histamine | positive |
| Purine metabolism | 71 | 2 | 0.03824 | 0.0873 | Adenosine 5'-diphosphate/AICAR | negative |

**Table S3. KEGG Co-Enrichment Analysis of Differential mRNAs and Differential Metabolites in Periodontitis with Hypertension Compared to the Healthy Group**

| **Pathway name** | **Total** | **Hits** | **P-value** | **Impact** | **Gene and Metabolism** | **Polarity mode** |
| --- | --- | --- | --- | --- | --- | --- |
| Phenylalanine, tyrosine and tryptophan biosynthesis | 11 | 3 | 0.003363 | 2.2 | L-Tyrosine; Tat; Got1 | positive |
| Phenylalanine metabolism | 24 | 4 | 0.004619 | 0.47826 | L-Tyrosine; Tat; Got1; Ddc | positive |
| Arginine biosynthesis | 27 | 4 | 0.006783 | 0.34615 | Ornithine; Nos1; LOC497963（Nos2）; Got1 | negative |
| Arginine and proline metabolism | 78 | 6 | 0.02334 | 0.15584 | Ornithine; Nos1; LOC497963(Nos2); Ckm; Ckmt2; Got1 | negative |

**Table S4. KEGG Enrichment Analysis of Differentially Expressed Genes in Periodontitis with Hypertension Compared to the Periodontitis Group (Top 10)**

| **KEGG ID** | **Description** | **GeneRatio** | **padj** | **geneName** |
| --- | --- | --- | --- | --- |
| rno04621 | NOD-like receptor signaling pathway | 8/35 | 0.000101 | Irf7/Defb4/Gbp5/Gbp4/Gbp2/AABR07013651.1/Defb5/Irf9 |
| rno05164 | Influenza A | 7/35 | 0.000215 | Rsad2/Irf7/Mx1/Mx2/RT1-Ha/Eif2ak2/Irf9 |
| rno05160 | Hepatitis C | 7/35 | 0.000215 | Rsad2/Irf7/Cldn17/Mx1/Mx2/Eif2ak2/Irf9 |
| rno05165 | Human papillomavirus infection | 8/35 | 0.00349 | Oasl2/Isg15/Mx1/Mx2/Oasl/RT1-M2/Eif2ak2/Irf9 |
| rno05162 | Measles | 5/35 | 0.009243 | Irf7/Mx1/Mx2/Eif2ak2/Irf9 |
| rno05169 | Epstein-Barr virus infection | 6/35 | 0.009243 | Isg15/Irf7/RT1-Ha/RT1-M2/Eif2ak2/Irf9 |
| rno05203 | Viral carcinogenesis | 5/35 | 0.0528 | Irf7/RT1-M2/Eif2ak2/Hdac6/Irf9 |
| rno05150 | Staphylococcus aureus infection | 3/35 | 0.079861 | Defb4/RT1-Ha/Defb5 |
| rno05167 | Kaposi sarcoma-associated herpesvirus infection | 4/35 | 0.195848 | Irf7/RT1-M2/Eif2ak2/Irf9 |
| rno05171 | Coronavirus disease - COVID-19 | 6/35 | 0.198647 | Isg15/Ace2/Mx1/Mx2/Eif2ak2/Irf9 |

**Table S5. KEGG Enrichment Analysis of Differential Metabolites in Periodontitis with Hypertension Compared to the Periodontitis Group**

| **pathway name** | **Total** | **Hits** | **P-value** | **Impact** | **Metabolism** | **Polarity mode** |
| --- | --- | --- | --- | --- | --- | --- |
| Arginine biosynthesis | 14 | 2 | 0.000436 | 0.28934 | Citrulline，Ornithine | negative |
| Glutathione metabolism | 28 | 1 | 0.069301 | 0 | Ornithine | negative |
| Arginine and proline metabolism | 36 | 1 | 0.088423 | 0.16395 | Ornithine | negative |
| Pyrimidine metabolism | 39 | 1 | 0.095517 | 0.0172 | CDP | negative |
| Drug metabolism - cytochrome P450 | 55 | 1 | 0.13265 | 0 | Valproic acid | negative |

**Table S6. KEGG Co-Enrichment Analysis of Differential mRNAs and Differential Metabolites in Periodontitis with Hypertension Compared to the Periodontitis Group**

| **Pathway name** | **Total** | **Hits** | **P-value** | **Impact** | **Gene and Metabolism** | **Polarity mode** |
| --- | --- | --- | --- | --- | --- | --- |
| Arginine biosynthesis | 27 | 3 | 0.000104 | 0.38462 | Citrulline, Ornithine, Arg1 | negative |
| Arginine and proline metabolism | 78 | 2 | 0.032676 | 0.077922 | Ornithine, Arg1 | negative |

**Table S7.** **Primers for Gene Expression Detection**

| **Gene** | **Sequence (5>3)** | |
| --- | --- | --- |
| *IL6* | forward | CACTGGTCTTTTGGAGTTTGAG |
|  | reverse | GGACTTTTGTACTCATCTGCAC |
| *IL8* | forward | ACCTCAACCTCCCAGACTCAAGTG |
|  | reverse | CTAGGCAACATAGCAAGACCGTGTC |
| *IFIT2* | forward | GAAGACAAGGCCATCCACCA |
|  | reverse | CAGCTCCTGAAGGAATGCCA |
| *IFIT3* | forward | ACCAGATTGGGTGCTGCTAC |
|  | reverse | GGAACTCAGCGAGATCGGAG |
| *ISG15* | forward | GTGGACAAATGCGACGAACC |
|  | reverse | TCGAAGGTCAGCCAGAACAG |
| *USP18* | forward | GGCTCCTGAGGCAAATCTGT |
|  | reverse | GGCACCGTGATCCTCTTCAA |
| *IRF7* | forward | GCTCCCCACGCTATACCATC |
|  | reverse | CAGGGAAGACACACCCTCAC |
| *MX1* | forward | CTCCGACACGAGTTCCACAA |
|  | reverse | GGCTCTTCCAGTGCCTTGAT |
| GAPDH | forward | CTTTGGTATCGTGGAAGGACTC |
|  | reverse | CAGTAGAGGCAGGGATGATGTT |
